# Molecular Evidence for the Parallel Evolution of Anosmia in Multiple Odontoceti (Toothed Whales) Clades

**DOI:** 10.3390/genes17060651

**Published:** 2026-05-31

**Authors:** Mark S. Springer, Michael R. McGowen, John Gatesy

**Affiliations:** 1 Department of Evolution, Ecology, and Organismal Biology, University of California, Riverside, CA 92521, USA; 2 Department of Vertebrate Zoology, Smithsonian National Museum of Natural History, MRC 108, P.O. Box 37012, Washington, DC 20013, USA; mcgowenm@si.edu; 3 Division of Vertebrate Zoology, American Museum of Natural History, New York, NY 10024, USA; johngatesy2@gmail.com

**Keywords:** anosmia, Odontoceti, olfaction, pseudogenes, toothed whales, Ziphiidae

## Abstract

**Background/Objectives.** Living odontocetes are the only extant mammals that lack anatomical features associated with olfaction. A leading hypothesis for the presumed complete loss of smell (i.e., anosmia) in odontocetes is that olfaction was lost in the common ancestor of the crown clade. An alternative hypothesis is that olfaction was lost in parallel in different odontocete lineages. **Methods.** A data set that includes complete coding sequences for three olfactory-specific genes (*CNGA2*, *CNGA4*, *OMP*) was assembled for 65 odontocete, 14 mysticete, and 28 outgroup species. The phylogenetic distribution of inactivating mutations in these genes was documented, and selection (dN/dS) analyses were performed with codeml to determine if selection intensity has been the same or different on three different branch categories within Odontoceti: intact, transitional, and fully pseudogenic. **Results.** There are no inactivating mutations that are shared by all odontocetes or either of the basal clades within Odontoceti. Fifty-four of 65 taxa exhibit at least one inactivating mutation, and all 11 species that lack inactivating mutations are ziphiids. Selection analyses indicate that dN/dS values are elevated on all branch categories within Odontoceti relative to both mysticete and outgroup branches. However, the odontocete dN/dS values are lowest on intact branches, intermediate on transitional branches, and highest on fully pseudogenic branches. **Conclusions.** The results of selection analyses, coupled with the absence of inactivating mutations on the most basal odontocete branches, support the hypothesis that olfaction was gradually reduced across Odontoceti prior to the complete ablation of this sense in independent lineages within this clade.

## 1. Introduction

Olfaction is an ancient sensory system that was present in the common ancestor of vertebrates and allowed for the detection of waterborne odorants [1]. Indeed, vertebrate-like olfactory receptors can be traced as far back as the origin of Chordata [2]. Subsequent to the origin of the first vertebrates, the transition from water to land resulted in key differences between the olfactory receptor repertoires of fishes and terrestrial vertebrates [3]. Specifically, the ability to detect airborne odorants is greatly expanded in tetrapods [4]. Extant lungfish, which are the closest living relatives to Tetrapoda, also exhibit significant expansion of their olfactory receptor repertoire for airborne odorants [5].

In most mammals, the olfactory receptor component of the genome is an order of magnitude larger than in many other vertebrates [6,7]. This subgenome is associated with extensive modifications at the anatomical level. Rowe et al. [7] proposed that the evolution of the mammalian olfactory system occurred in three distinct pulses. Changes that occurred in the first pulse are evidenced in *Morgonucodon* (~205 million years ago), which is a basal member of Mammaliaformes. *Morganucodon* has an encephalization quotient that is ~50% higher than that of basal cynodonts and most of the increase in brain size is accounted for by expansion of the olfactory bulbs and olfactory cortex. The second pulse of encephalization is marked by *Hadrocodium* (~195 million years ago), which is a taxon that is more crownward on the mammalian stem. As in the first pulse, the main areas of expansion were the olfactory bulbs and olfactory cortex. The final pulse of evolution occurred in the ancestor of crown Mammalia and was highlighted by ossification of the ethmoid turbinals to form the cribriform plate and a rigid scaffold in the nasal cavity to support the olfactory epithelium [7]. This epithelium includes olfactory receptor neurons that express a wide array of olfactory receptor (OR) genes. OR genes comprise the largest gene family in terrestrial mammals, and there are ~2000 intact (=functional) OR genes in the African elephant genome [8]. At the other extreme, there are fewer than 400 intact olfactory receptor genes in some primates and bats [9,10].

In contrast with most terrestrial mammals, living cetaceans have either reduced or lost their sense of smell. The medical terms for these conditions in humans are hyposmia (reduced smell) and anosmia (absent smell) [11]. Unlike our own species where these conditions can have a negative impact on health and quality of life [11,12], the reduced/absent sense of smell among different cetacean species is perhaps expected given that the mammalian olfactory system for detecting airborne odors is of limited utility for mammals that are entirely aquatic. Extant mysticetes that have been investigated retain morphological structures that are consistent with a functional, albeit reduced, sense of smell. These include paired ethmoturbinates and a cribriform plate with multiple foramina on each side of the plate as in terrestrial mammals [13,14,15,16,17]. Berta et al. [14] suggested that ethmoturbinates in mysticetes are presumably covered with an olfactory epithelium. More recently, Farnkopf et al. [17] provided histological evidence for the presence of an olfactory epithelium in *Balaena mysticetus* (bowhead whale) that covers at least part of the ethmoturbinates and further demonstrated that the olfactory epithelium expresses olfactory marker protein (OMP). This protein is only expressed in mature olfactory sensory neurons [17]. More recently, Hirose et al. [18] demonstrated that OR genes are expressed in the nasal mucosa of *Balaenoptera acutorostrata* (common minke whale). Mysticetes also retain olfactory bulbs, which further implies that baleen whales have a sense of smell [19,20]. The presence of a functional sense of olfaction in mysticetes may be useful for detecting the airborne odors derived from planktonic invertebrates [21,22].

Extant adult odontocetes that have been examined have lost or reduced all three components of the ethmoid (perpendicular plate, lateral mass [ectethmoids] that includes the ethmoturbinates, a perforated cribriform plate) relative to the condition observed in mysticetes [14]. Ichishima [16] examined representative odontocetes belonging to Delphinidae (five species), Ziphiidae (several species), and Phocoenidae (one species) and found no trace of the ethmoturbinates, which support the olfactory epithelium in other mammals. Odontocetes appear to retain a cribriform plate, but there is no consensus on whether this plate contains perforations for transit of branches of the olfactory nerve. Mead and Fordyce [23] suggested that perforations are absent in odontocetes, but also noted that perforations in the cribriform plate were reported in *Physeter* [24] and *Berardius* [25]. More recently, Farnkopf et al. [26] reported the presence of one or more perforations in the cribriform plate of four extant odontocetes (*Delphinapterus leucas*, *Kogia breviceps*, *Phocoena phocoena*, *Tursiops truncatus*) that were sampled. Computerized tomography (CT) reconstructions of the cribriform plate of *D. leucas* show that there are several foramina in this species, although it remains unclear if these are passages for vasculature, remnants of the olfactory nerve, or remnants of the terminal nerve [26]. Key components of the nervous system that mediate olfaction (e.g., olfactory nerve, olfactory bulb, olfactory tract) are present in early developmental stages of odontocetes, but atrophy in adult toothed whales that have been studied [26,27,28,29,30].

The continued reduction and even the complete loss of olfaction in odontocetes that have been investigated may have been facilitated by the prior evolution of echolocation, i.e., the echolocation priority hypothesis [31]. Indeed, echolocation evolved on the stem odontocete branch, earlier than the most recent common ancestor of crown Odontoceti, based on a cladistic analysis of fossil and extant cetaceans [32]. Odontocetes also share numerous amino acid changes in the hearing protein prestin that are essential for echolocation and presumably originated in the common ancestor of this clade [33]. Importantly, echolocation in living odontocetes is an important sense for hunting prey, and along with vision may have obviated any need for the detection of airborne odors in prey detection [14,21]. However, it remains unclear if olfaction was also lost on the stem odontocete branch [34,35] or if anosmia evolved convergently in different odontocete lineages [36,37]. The latter hypothesis is more viable in view of a recent phylogeny [38] that positions several extinct odontocetes with anatomical proxies for olfaction (albeit reduced) within crown Odontoceti [37].

At the molecular level, both mysticetes and odontocetes have reduced repertoires of functional OR genes, and this reduction is more pronounced in odontocetes. Kishida et al. [20] estimated that there are 12 intact OR genes in the *Tursiops truncatus* (bottlenose dolphin) genome in comparison to 60 functional OR genes in *Balaenoptera bonaerensis* (Antarctic minke whale). Data from the chordate olfactory receptor database (CORD) [39,40] also support the conclusion that mysticetes have more functional OR genes than odontocetes. The mean number of functional OR genes for seven mysticetes in the CORD is ~83.1, whereas the mean for 21 odontocetes is only ~31.6 functional OR genes. Finally, Farnkopf et al. [26] estimated that two mysticetes have 79–80 intact OR genes, whereas four odontocetes have 6–21 intact OR genes. Farnkopf et al. [26] also found that the number of intact OR genes is correlated with the surface area of the cribriform plate (body size-corrected) in cetartiodactyl taxa. These authors estimated the surface area of the cribriform plate in several fossil cetaceans and used their regression results to predict the size of the OR repertoire in these extinct taxa. A key result that emerged from this study is that a substantial loss of OR genes occurred during the early/middle Eocene in archaeocete ancestors before cetaceans became obligately aquatic. The pakicetid *Ichthyolestes pinfoldi* (~50 million years) was estimated to have 731–766 intact OR genes, and the remingtonocetid *Remingtonocetus harudiensis* (~42 million years) was estimated to have 307–333 intact genes. Farnkopf et al. [26] also provided an estimate of 183–201 intact OR genes for the Miocene squalodontid *Squalodon murdochi* (16 million years). The olfactory anatomy of this taxon includes ethmoturbinates [13], and multiple cladistic analyses place squalodontids on the odontocete stem, e.g., [38,41]. The final extinct taxon for which Farnkopf et al. [26] estimated the number of intact OR genes is the crown odontocete *Isthminia panamensis* (6 million years), which is the sister taxon to the extant river dolphin *Inia geoffrensis* in Pyenson et al.’s [42] cladistic analysis. Farnkopf et al. [26] estimated that there were 83–93 intact genes in *I. panamensis*. This taxon also has at least two foramina (1 mm in diameter) in the cribriform plate that communicate between the cranial cavity and the nasal passage [26]. Among extant odontocetes, the maximum number of intact OR genes reported by Farnkopf et al. [26] is 21 in *Delphinapterus*, which is also the odontocete taxon with the largest cribriform plate and the greatest number of perforations in this anatomical structure. Given their results, Farnkopf et al. [26] even suggested that *Delphinapterus* may retain some form of olfaction to detect predators (polar bears), while at the same time acknowledging this taxon does not have olfactory turbinates or a macroscopic olfactory bulb.

Importantly, the loss and pseudogenization of numerous OR genes in Odontoceti may indicate a loss of sensitivity to specific odorants rather than total anosmia [36,43]. By contrast, the inactivation of single-copy genes in the olfactory signal transduction cascade would provide more compelling evidence for the loss of functional olfaction. Springer and Gatesy [36] examined three olfactory-specific genes (*CNGA2*, *CNGA4*, *OMP*) in representative odontocetes and documented the occurrence of inactivating mutations in all three genes. However, taxon sampling was very limited, especially for the cyclic nucleotide-gated (CNG) channel genes for which there were only four odontocetes. Also, Springer and Gatesy [36] did not find any inactivating mutations that are shared by all odontocetes, suggesting that anosmia may have evolved convergently within Odontoceti. More recently, Jauhal et al. [44] examined coding sequences for nine olfactory-related genes in seven odontocetes, eight mysticetes, and three non-cetacean outgroups. They reported inactivating mutations in five genes, but none were shared by all seven odontocetes (*Lipotes*, two monodontids, four delphinids) that were examined. Jauhul et al. [44] also performed selection analyses to determine if there are significant differences in dN/dS values on four categories of branches (outgroups, stem Cetacea, Mysticeti, Odontoceti), although these authors did not specifically address the hypothesis that anosmia may have evolved convergently within Odontoceti.

In the present study, taxonomic coverage for the three single-copy olfactory genes (*CNGA2*, *CNGA4*, *OMP*) examined in Springer and Gatesy [36] was expanded to include the majority of odontocete species (65 of 79) (https://marinemammalscience.org/science-and-publications/list-marine-mammal-species-subspecies/; accessed 20 February 2026) and two competing hypotheses for anosmia in odontocetes were tested: (1) anosmia evolved just once in the common ancestor of Odontoceti versus (2) anosmia evolved independently in two or more different odontocete lineages. Multiple types of inactivating mutations in these genes were documented and mapped onto a species tree for Odontoceti, and selection (dN/dS) analyses were performed to determine whether purifying selection has been relaxed equivalently on functional (intact) Odontoceti branches, transitional branches that record the first inactivating mutation in a clade, and fully pseudogenic branches that post-date an earlier mutation on a transitional branch. Finally, these results are discussed in the context of recent paleontological and behavioral evidence bearing on the occurrence of anosmia, a feature that, to our knowledge, has only evolved in Odontoceti and possibly hydrophoiin sea snakes [4] in the entire history of vertebrates that spans more than 500 million years [45,46].

## 2. Materials and Methods

### 2.1. Gene Sampling and Rationale

Three olfactory-specific genes (*CNGA2*, *CNGA4*, *OMP*) that were previously shown to be inactivated in representative odontocetes [36] were targeted for further investigation with expanded taxon sampling. *CNGA2* and *CNGA4* encode two of the three subunits that comprise olfactory cyclic nucleotide-gated (CNG) ion channels. These channels are heterotetramers that include two subunits of the CNGA2 protein, one subunit of the CNGA4 protein, and one subunit of the CNGB1 protein. Of these, CNGA2 is the principal subunit of its CNG channel [47]. CNGA4, in turn, is a modulating subunit of olfactory CNG channels that is required for odor adaptation [47]. Unlike CNGA2 and CNGA4, which are thought to be olfactory specific, CNGB1 is pleiotropic and is also associated with the rod phototransduction cascade [48]. Olfactory CNG channels are critical players in olfactory signal transduction, and functional channels are required for the proper depolarization and relay of action potentials to the brain following the binding of an odorant molecule to an olfactory neuron [49]. *CNGA2*-knockout mice are congenitally anosmic [50,51]. Furthermore, two different inactivating mutations in the human *CNGA2* gene are known to cause anosmia [51,52]. Mice that are deficient in *CNGA4*, in turn, show defects in odor adaptation [53,54]. More specifically, the CNGA4 subunit accelerates Ca2^+^ mediated negative feedback in olfactory signaling that allows for rapid adaptation of this sensory system [53]. Finally, the *OMP* gene encodes a protein that plays an important role in the transduction of olfactory signals. More specifically, it may regulate the kinetics and termination of olfactory responses and facilitate the temporal resolution of odor stimulus [55]. *OMP*-knockout mice are compromised in their ability to respond to odors and have reduced neural activity projected toward the olfactory bulb [56].

### 2.2. Taxon Sampling

Taxon sampling included 107 species, of which 79 are cetaceans and 28 are outgroups to Cetacea. Taxon sampling for Cetacea included 65 odontocete species and 14 mysticete species. Cetacean sampling encompasses representatives of every extant family and all genera except for *Sotalia* (family Delphinidae) and *Phocoenoides* (family Phocoenidae). Outgroups included 26 non-cetacean cetartiodactyls and two perissodactyls. Individual species are listed in Table 1.

### 2.3. BLAST Searches and Procurement of Molecular Data

DNA sequences for three olfactory-specific genes (*CNGA2*, *CNGA4*, *OMP*) were obtained from both assembled genomes and unassembled sequence reads. Assembled genomes were sourced at NCBI (https://www.ncbi.nlm.nih.gov/, accessed on 20 March 2026), DNA Zoo (https://www.dnazoo.org/, accessed on 1 August 2024 and 15 June 2025) [57], and The Bowhead Whale Genome Resource (http://www.bowhead-whale.org/, accessed on 20 February 2024) [58]. Unassembled sequence reads were procured from NCBI’s Sequence Read Archive (SRA, https://www.ncbi.nlm.nih.gov/sra, accessed on 20 March 2026) and from newly generated Illumina whole-genome sequence data (J.G., M.R.M., M.S.S.) (see Table S1 for accession numbers as well as institutional sample ID numbers for newly generated sequences). NCBI’s RefSeq and Nucleotide databases were searched with HUGO gene symbols (CNGA2, CNGA4, OMP) in conjunction with species names for representative ingroup (*Balaenoptera musculus*, *Orcinus orca*, *Tursiops truncatus*) and outgroup (*Bos taurus*, *Capra hircus*, *Camelus ferus*, *Equus caballus*) taxa. Sequences for each reference species were imported into Geneious Prime (version 2025.1.2, https://geneious.com) [59]. Gene sequences were aligned with MAFFT [60,61,62]. Sequences for additional species were obtained with NCBI’s Nucleotide Basic Local Alignment Search Tool (BLAST). BLAST+ 2.17.0 was used to search assembled genomes in the “RefSeq Genome Database” and “Whole-genome shotgun contigs” database. BLAST was also used to search assembled genomes that were downloaded from DNA Zoo and imported into Geneious Prime. Finally, BLAST was used to search unassembled genomes in the SRA database. Query sequences for BLAST searches were obtained from closely related species. MegaBLAST was used for highly similar sequences, i.e., taxa in the same genus or family. Blastn was used for more divergent sequences from taxa in different families. The highest-scoring BLAST results from NCBI database searches were imported into Geneious Prime. Sequences that were extracted from the SRA database were assembled using Geneious Prime’s “Map to Reference” approach, where the reference sequence was a closely related species to the SRA taxon. The maximum mismatch was set at 10% per read and a minimum of two reads was required for base calling with a consensus threshold of 65%. The map to reference approach in Geneious Prime was also used to obtain *CNGA2*, *CNGA4*, and *OMP* sequences for 33 odontocete species and one mysticete species (Table S1) for which whole-genome DNA libraries were constructed and paired-end Illumina sequence reads were generated (see Section 2.4 below). As above, the maximum allowable mismatch was set at 10% per read with a closely related reference taxon, and a minimum of two reads was required for base calling with a consensus threshold of 65%. A total of 98 new gene sequences were generated from the newly generated Illumina whole-genome libraries.

### 2.4. Library Construction and DNA Sequencing

DNA samples for 34 cetacean species were used to construct whole-genome DNA libraries. Of these, 29 samples were provided by the Southwest Fisheries Science Center and five samples were provided by the Smithsonian National Museum of Natural History. DNA libraries for the majority of odontocete samples were constructed with Illumina’s NeoPrep procedure following sonication of the genomic DNA samples to a mean length of ~550 bp at the Genomic Core Facility at the University of California, Riverside (UCR). Libraries for four species of *Mesoplodon* (*M. bowdoini*, *M. ginkgodens*, *M. grayi*, *M. layardii*) were constructed with a NEB Ultra II DNA kit with dual unique indexes. Lastly, NEBNext Ultra DNA Library Prep Kits were used to construct Illumina DNA libraries for five odontocete species (*Hyperoodon ampullatus*, *Lagenorhynchus albirostris*, *Mesoplodon mirus*, *M. perrini*, *M. stejnegeri*) at the Smithsonian National Museum of Natural History (NMNH) Laboratory of Analytical Biology. All of the DNA libraries except for the NEB Ultra II libraries (four *Mesoplodon* spp.) were sequenced at ~30–45× coverage at the New York Genome Center using paired-end sequencing (150 bp per read) on a HiSeq 2500 platform. The NEB Ultra II DNA libraries for the four *Mesoplodon* species were sequenced at the same coverage on a NovaSeq platform with paired-end sequencing (150 bp per read).

### 2.5. Alignments and Inactivating Mutations

Complete protein-coding sequences and introns were aligned with MAFFT in Geneious Prime and manually adjusted by eye. Alignments were inspected for the following types of inactivating mutations: start codon mutations, premature stop codons, termination codon mutations (i.e., that eliminate the canonical stop codon), frameshift insertions and deletions, intron–exon and exon–intron boundary deletions, and splice site mutations. Inactivating mutations were mapped onto a species tree for cetaceans (see Section 2.7 below) with both accelerated transformation (AccTran) and delayed transformation (DelTran) character optimization methods with PAUP 4.0a [63]. AccTran favors reversals over parallelisms, whereas DelTran favors parallelisms over reversals.

### 2.6. Gene Trees

Maximum likelihood (ML) gene trees for *CNGA2*, *CNGA4*, and *OMP* were constructed from aligned protein-coding sequences, including frameshift insertions, with RAxML 8.2.11 [64] and a general time-reversible model of sequence evolution with an allowance for among-site rate variation as implemented in Geneious Prime. Tree searches were performed with 500 pseudoreplications of rapid bootstrapping followed by a search for the best-scoring ML tree.

### 2.7. Selection Analyses

Selection analyses were performed with a species tree that was compiled from McGowen et al. [65] for most cetacean relationships, Meredith et al. [66] for interfamilial and higher relationships among non-cetacean outgroups, and Hassanin et al. [67] for intrafamilial relationships among non-cetacean cetartiodactyls. Eleven odontocete species that were absent from McGowen et al. [65] were positioned on the species tree based on Rosel et al. [68] (*Balaenoptera ricei*), McGowen et al. [69] (*Cephalorhynchus eutropia*, *Cephalorhynchus hectori*, *Phocoena sinus*, *Platanista minor*), Zurano et al. [70] (*Indopacetus pacificus*), Yamada et al. [71] (*Berardius minimus*), and Chehida et al. [72] (*Neophocaena asiaeorientalis*). Selection analyses were performed with the codeml program in PAML 4.9 and the branch model [73]. Analyses were performed with codon frequency model 1 (CF1) and codon frequency model 2 (CF2). Akaike Information Criterion (AIC) scores were used to select the most appropriate codon frequency model (CF1 or CF2) for each of three olfactory-specific genes (*CNGA2*, *CNGA4*, *OMP*) and a concatenation of these genes. Six different branch categories were employed based on anatomical features of crown cetaceans and patterns of inactivation among the three olfactory-specific genes. The six categories are as follows: (1) branches that lead to perissodactyls and to non-cetacean cetartiodactyls, all of which have intact olfactory anatomy and functional copies of the three single-copy, olfactory-specific genes; (2) the stem Cetacea branch, on which the olfactory bulb may have been reduced given that it is absent or greatly reduced in all crown cetaceans that have been investigated; (3) stem and crown Mysticeti branches given that mysticetes retain a functional albeit highly reduced olfactory bulb; (4) odontocete branches that retain intact copies (i.e., no inactivating mutations) of all three olfactory-specific genes; (5) all fully pseudogenic branches that post-date the occurrence of an inactivating mutation in at least one of the olfactory-specific genes; and (6) 16 transitional branches that record the first inactivating mutation(s) in a species or clade as follows: *Physeter macrocephalus*, stem *Kogia*, stem *Platanista*, *Indopacetus pacificus*, *Mesoplodon bidens*, *M. europaeus*, *M. carlhubbsi, M. bowdoini*, *M. hectori*, *M. stejnegeri*, *M. densirostris*, *M. perrini*, *Lipotes vexillifer*, *Inia geoffrensis*, *Pontoporia blainvillei*, stem Delphinoidea (Delphinidae + Monodontidae + Phocoenidae). Selective pressures may be heterogeneous within the transitional branch category, but the dN/dS value for the combined transitional branch category is expected to be intermediate between dN/dS values for the intact branches category and the fully pseudogenic branches category if anosmia evolved independently in different odontocete clades. DelTran optimizations of inactivating mutations were used to discriminate among different branch categories.

Selection analyses were also performed on *CNGA2* and *CNGA4* after eliminating all codons with CpG dinucleotides, either in a single codon or in adjacent codons, in the most recent common ancestor of Odontoceti. CpG sites are regions of a DNA strand where a cytosine nucleotide occurs next to a guanine nucleotide in the linear 5′ to 3′ sequence of bases along its length. The opposite strand will also have a 5′ to 3′ CpG dinucleotide at the same location. These analyses were performed to test the hypothesis that CpG sites, which have elevated substitution rates from C to T when cytosine is methylated [74], are disproportionately located at nonsynonymous sites in *CNGA2* and *CNGA4* and may cause the elevated dN/dS ratios that were observed in these genes (see Section 3.4 below).

## 3. Results

### 3.1. Alignments and Gene Trees

**Figure 1:**
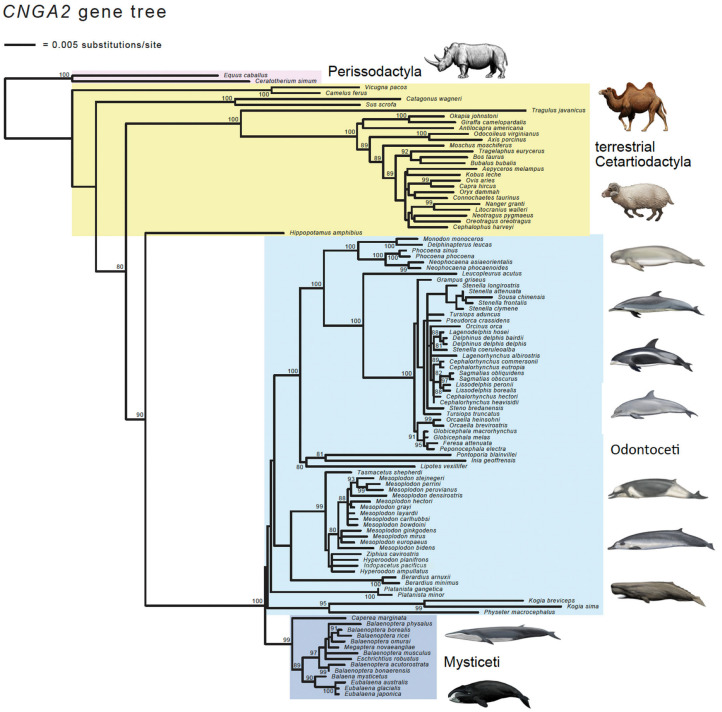
Maximum likelihood (RAxML) gene tree with bootstrap support values for *CNGA2*. Only bootstrap support values ≥ 80% are shown. See Newick tree files in Supplementary Materials for support values that are <80%.

**Figure 2:**
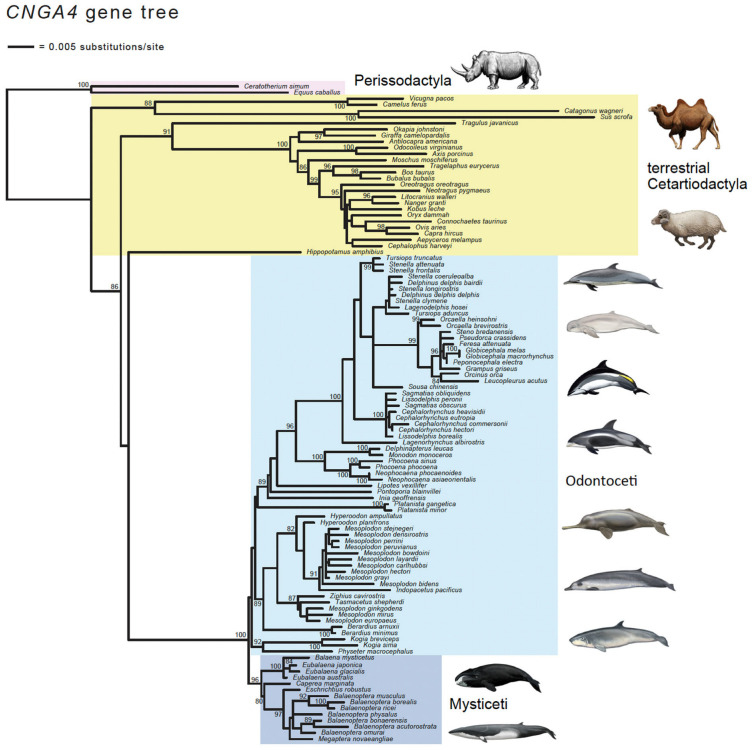
Maximum likelihood (RAxML) gene tree with bootstrap support values for *CNGA4*. Only bootstrap support values ≥ 80% are shown. See Newick tree files in Supplementary Materials for support values that are <80%.

**Figure 3:**
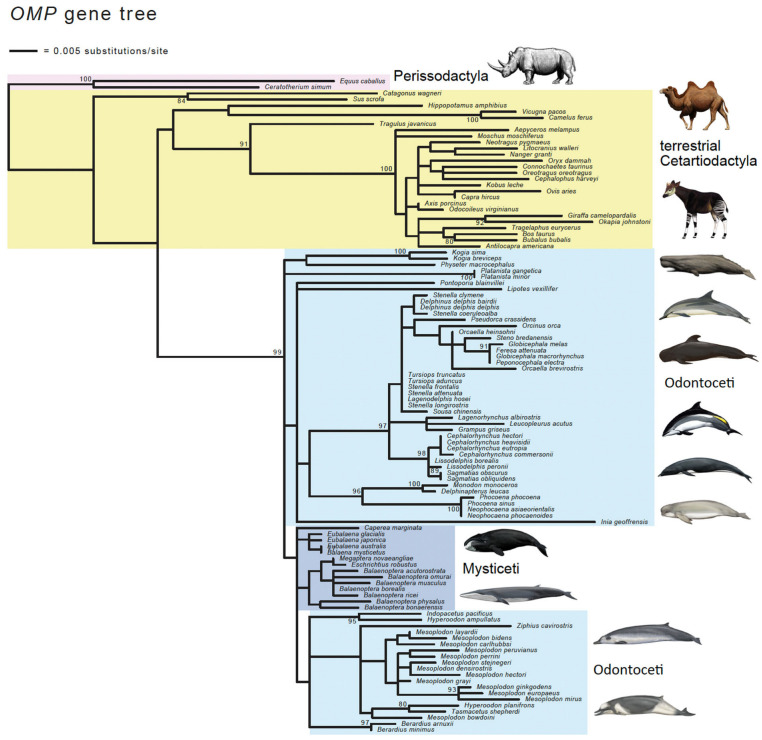
Maximum likelihood (RAxML) gene tree with bootstrap support values for *OMP*. Only bootstrap support values ≥ 80% are shown. See Newick tree files in Supplementary Materials for support values that are <80%.

The lengths of the coding sequence alignments, inclusive of frameshift insertions, are as follows: *CNGA2* = 2019 bp; *CNGA4* = 1749 bp; *OMP* = 532 bp. The lengths of the alignments for selection analyses with codeml, which excluded alignment positions with frameshift insertions and termination codons, are all shorter as follows: *CNGA2* = 2001 bp; *CNGA4* = 1728 bp; *OMP* = 507 bp. Both sets of alignments are provided in the Supplementary Materials. Maximum likelihood (RAxML) trees are shown in Figure 1, Figure 2 and Figure 3 and Newick versions of these trees are provided in the Supplementary Materials. All three of the individual gene trees exhibit minor conflicts with McGowen et al.’s [65] well-supported species tree that is based on ~3200 protein-coding genes, but such conflicts are expected for short sequences because of gene-tree reconstruction errors and incomplete lineage sorting that most commonly affect short internodes [75]. For example, ziphiids and platanistids are in inverted positions on the *CNGA4* gene tree (Figure 2) relative to the species tree [65] and odontocetes are paraphyletic on the *OMP* gene tree (Figure 3).

### 3.2. Inactivating Mutations

**Figure 4:**
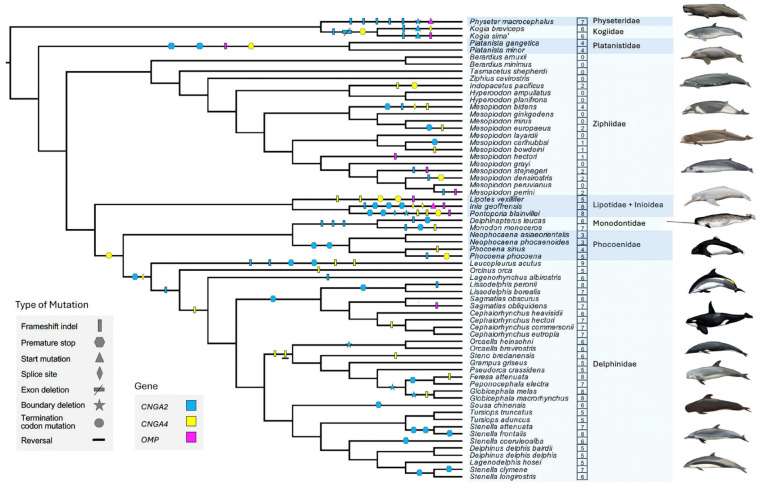
Delayed transformation (DelTran) character optimization of inactivating mutations on a species tree for Odontoceti.

**Figure 5:**
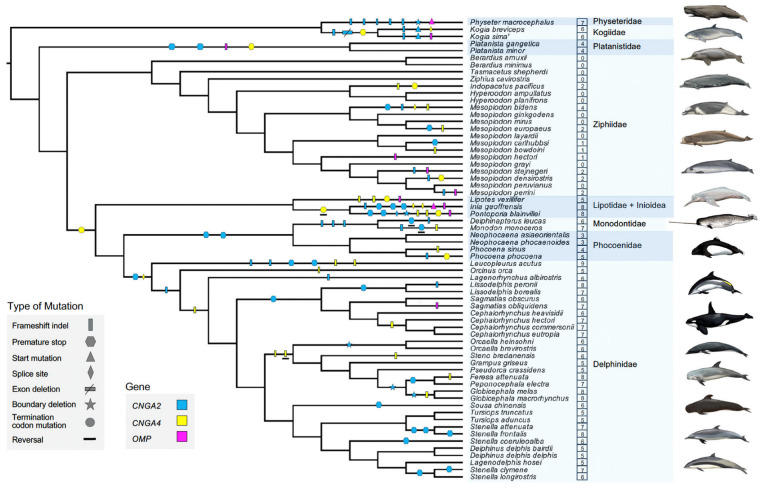
Accelerated transformation (AccTran) character optimization of inactivating mutations on a species tree for Odontoceti.

**Figure 6:**
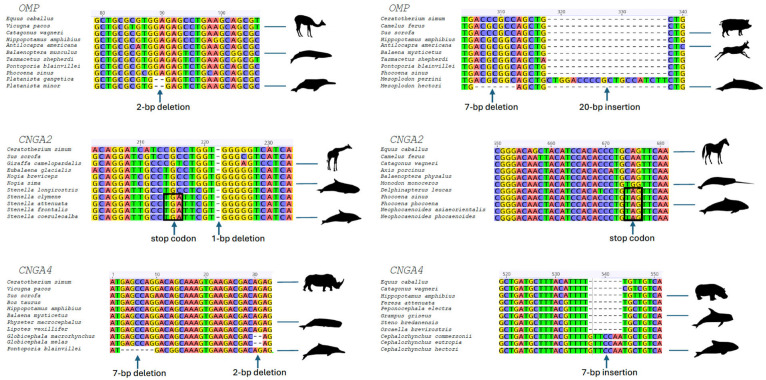
Examples of inactivating mutations in three olfactory-specific genes (*OMP*, *CNGA2*, *CNGA4*) in Odontoceti.

Inactivating mutations were mapped onto a species tree for Cetacea with both DelTran (Figure 4) and AccTran (Figure 5) character optimization. These mutations are also enumerated in Table 2. Representative inactivating mutations are illustrated in Figure 6. Overall, there are more inactivating mutations in *CNGA2* (DelTran = 60; AccTran = 58), which is the longest coding sequence (2006 bp with termination codon), than in *CNGA4* (DelTran = 33; AccTran = 32), which has an intermediate length (1731 bp with termination codon), or *OMP* (11 with both optimization methods), which is the shortest sequence (510 bp with termination codon). The observed number of inactivating mutations in each gene based on length of the coding sequences is not significantly different from expectations with a Chi-square test (AccTran: *p* = 0.11, df = 2; DelTran: *p* = 0.10, df = 2). The most common inactivating mutations (DelTran mapping) are frameshift indels (*n* = 52), of which 21 are frameshift insertions and 31 are frameshift deletions. Premature stop codons are also common (*n* = 34). Other inactivating mutations have occurred less frequently: splice site mutations = 6; boundary deletions (intron–exon or exon–intron) = 5; start codon mutations = 4, termination codon mutations = 2; whole exon deletions = 1.

DelTran mapping resulted in 104 inactivating mutations with one reversal whereas AccTran mapping yielded 101 inactivating mutations with four reversals. Nine inactivating mutations are homoplastic, of which six have identical reconstructions with both AccTran and DelTran (Table 2). Five of the identical reconstructions involve convergent occurrences of an inactivating mutation in two or more lineages: (1) an intron–exon boundary deletion (*CNGA2*) in *Orcaella* and *Feresa* + *Peponocephala* + *Globicephala*, (2) a premature stop codon (*CNGA2*) in *Platanista* and *Mesoplodon carlhubbsi*, (3) a premature stop codon (*CNGA2*) in *Stenella clymene*, *S. coeruleoalba*, and *S. attenuata + S. frontalis*; (4) a premature stop codon (*CNGA2*) in *Leucopleurus* and *Pontoporia*; and (5) a frameshift insertion (*CNGA4*) in *Leucopleurus*, *Orcinus*, and *Orcaella* + *Steno* + *Grampus* + *Pseudorca* + *Feresa* + *Peponocephala* + *Globicephala*. Among inactivating mutations with reversals, both optimization methods revealed a 1 bp deletion in exon 4 of *CNGA4* that originated in the common ancestor of all delphinids except for *Leucopleurus* and *Orcinus* that was reversed in the common ancestor of *Orcaella* + *Steno* + *Grampus* + *Pseudorca* + *Feresa* + *Peponocephala* + *Globicephala*. In addition, DelTran reconstructed the independent evolution of a premature stop codon in exon 5 of *CNGA2* in Phocoenidae and *Monodon*. This mutation was reconstructed to have originated in the common ancestor of Monodontidae + Phocoenidae with subsequent loss in *Delphinapterus* by AccTran. There is also a premature stop codon in exon 6 of *CNGA2* that was reconstructed to have evolved independently in Phocoenidae and *Delphinapterus* with DelTran, whereas AccTran reconstructed this mutation to have evolved in the common ancestor of Monodontidae + Phocoenidae with subsequent loss in *Monodon*. Finally, DelTran reconstructed the independent evolution of a termination codon mutation (GGA) in *CNGA4* in Delphinoidea and in *Lipotes* whereas AccTran reconstructed the origin of this mutation in Delphinida (=Delphinoidea + *Lipotes* + Inioidea) with a subsequent reversal in Inioidea (Iniidae + Pontoporiidae). The termination codon mutation results in an exon 6 length of 512 bp (inclusive of termination codon) in taxa with this mutation. With the exceptions of *Litocranius walleri* (428 bp) and *Sus scrofa* (509 bp), exon 6 of *CNGA4* is 461 bp in the non-cetaceans and mysticetes in that were sampled in this study. *L. walleri* possesses an in-frame 33 bp deletion that is 28 bp upstream of the termination codon. *S. scrofa*, in turn, has a 1 bp deletion that is 14 bp upstream of the TGA stop codon. This results in a termination codon that is 49 bp downstream from the canonical termination codon in other taxa.

Among the 65 odontocete species that were included in this study, the mean number of inactivating mutations (shared plus autapomorphic) is ~4.45 and the maximum number of mutations is nine in *Leucopleurus acutus*. At least one inactivating mutation is present in 54 of 65 odontocete species, and all 11 species that lack inactivating mutations are ziphiids. The mean number of inactivating mutations among ziphiid species (N = 20) is 0.85 and all 17 of the mutations are autapomorphic. Ziphiids without inactivating mutations include both species of *Berardius*, *Tasmacetus*, *Ziphius*, both species of *Hyperoodon*, and five species of *Mesoplodon*. By contrast, there are 17 synapomorphic mutations within Delphinida versus 42 (AccTran) or 44 (DelTran) autapomorphic inactivating mutations within this clade. The mean number of inactivating mutations in species belonging to Delphinida is ~6.36. Physeteroids have a similar number of inactivating mutations per species at ~6.33. Finally, there are four shared mutations and no autapomorphic mutations in *Platanista*.

Notably, there are no inactivating mutations that are shared by all odontocetes. Mutations are also absent on the two most basal branches within Odontoceti, stem Physeteroidea and stem Synrhina (i.e., non-physeteroid odontocetes) [41]) and on the stem branch to Ziphiidae + Delphinida. The most inclusive inactivating mutation is the previously mentioned termination codon mutation (*CNGA4*) in the common ancestor of Delphinida (AccTran) or Delphinoidea (DelTran). There are also shared inactivating mutations in Monodontidae + Phocoenidae (AccTran only), Monodontidae, Phocoenidae (DelTran only), Delphinidae, 12 clades within Delphinidae, Kogiidae, and Platanistidae.

### 3.3. Selection Analyses with All Codons

AIC scores were used to select the preferred codon frequency model (CF1 or CF2) in codeml, and the preferred model was CF2 for all three genes as well as the concatenation of these genes (Supplementary Table S2). dN/dS values for six different categories are provided in Table 3 for *CNGA2*, *CNGA4*, *OMP*, and a concatenation of all three genes. dN/dS values for non-cetacean branches are consistent with strong purifying selection: *CNGA2*: ~0.10; *CNGA4*: ~0.10; *OMP*: ~0.13; concatenation: ~0.11. Values for the stem Cetacea branch are higher than for the non-cetacean branches: *CNGA2*: ~0.24; *CNGA4*: ~0.32; *OMP*: ~0.18; concatenation: ~0.28. Values for the mysticete branches are similar to those for the stem Cetacea branch: *CNGA2*: ~0.45; *CNGA4*: ~0.24; *OMP*: ~0.13; concatenation: ~0.31. These values suggest that purifying selection has been less intense on the stem Cetacea and mysticete branches than non-cetacean branches. dN/dS values for the odontocete branches with an intact set of three genes are higher than for the mysticete branches: *CNGA2*: ~0.82; *CNGA4*: ~0.71; *OMP*: ~0.35; concatenation: ~0.72. Transitional branches, in turn, have higher dN/dS values than intact odontocete branches: *CNGA2*: ~1.14; *CNGA4*: ~0.87; *OMP*: ~0.48; concatenation: ~0.95. Finally, dN/dS values for the fully pseudogenic odontocete branches are higher than for the other branch categories: *CNGA2*: ~1.40; *CNGA4*: ~1.46; *OMP*: ~0.80; concatenation: ~1.39. Indeed, the dN/dS value for pseudogenic branches with the concatenation of three genes is almost twice as high as the value for the intact odontocete branches (~1.39 to ~0.72). A log-likelihood ratio test was used to compare the likelihood score of the concatenated analysis with three separate categories for the odontocete branches (intact, transitional, fully pseudogenic) to the likelihood score of an analysis where these three branch categories were combined into a single branch category. This test was used to evaluate the hypothesis that olfaction was completely ablated in the common ancestor of Odontoceti, in which case there should not be any differences in selection intensity among branch categories in this clade. The likelihood score for the analysis with separate categories was significantly better than the likelihood score for the analysis with a single category for the odontocete branches (*p* = 2.42 × 10^−5^, df = 2). This result suggests that different categories of odontocete branches have evolved under different selective pressures.

In the case of both cyclic nucleotide gated channel genes (*CNGA2*, *CNGA4*), dN/dS values for the fully pseudogenic category are >1, whereas values for the intact odontocete category are <1, suggesting that branches in the fully pseudogenic and intact odontocete categories may have evolved under positive selection and purifying selection (albeit relaxed), respectively. Furthermore, likelihood scores for analyses with an estimated value for the fully pseudogenic branch category were significantly better than likelihood scores for analyses with a fixed dN/dS value of 1.0 for the fully pseudogenic category for both *CNGA2* and *CNGA4*. While these results are suggestive of positive selection, other explanations for the elevated dN/dS values in the fully pseudogenic branch category in these two genes are possible and argue against positive selection (see Section 3.4 below). Finally, both fully pseudogenic and intact odontocete branches for the *OMP* gene have dN/dS values that are <1, although the value for the former category (~0.80) is more than 2× higher than the value for the latter category (~0.35). These results suggest both branch categories have evolved under purifying selection. However, there was not a significant difference between analyses with an estimated valued for the fully pseudogenic category (~0.80) versus a fixed valued of 1.0 for this category (*p* = 0.41). The *OMP* results are therefore consistent with neutral evolution in the fully pseudogenic category.

### 3.4. Selection Analyses Without CpG Codons

Selection analyses were performed on *CNGA2* and *CNGA4* protein-coding sequences after eliminating all codons with CpG dinucleotides, either in a single codon or in adjacent codons, in the most recent common ancestor of Odontoceti. These analyses allowed us to test the hypothesis that CpG sites are disproportionately located at nonsynonymous sites in *CNGA2* and *CNGA4* and may have resulted in the elevated dN/dS ratios (>1.0) in the fully pseudogenic branch category for these genes. *CNGA2* included 49 codons that incorporate all or part of a CpG dinucleotide in the common ancestor of Odontoceti, and *CNGA4* included 82 codons with all or part of a CpG dinucleotide. When these codons were excluded from selection analyses, the log likelihood scores with estimated and fixed (1.0) dN/dS values for the fully pseudogenic category were not significantly different from each other (*CNGA2*: *p* = 0.37; *CNGA4*: *p* = 0.06). These results are consistent with neutral evolution rather than positive selection.

## 4. Discussion

### 4.1. Patterns of Gene Inactivation and Neutral Evolution

If olfaction was ablated in the common ancestor of Odontoceti [34,35], then molecular evidence of this ablation may be expected in the form of shared inactivating mutations and/or neutral evolution in olfactory-specific genes that are essential for proper functioning of the olfactory signal transduction cascade. Among the three genes that were examined in this study, there are no inactivating mutations in the common ancestor of Odontoceti or on the basal branches within Odontoceti that lead to Physeteroidea and Synrhina. The most inclusive mutation that includes multiple odontocete families is a termination codon mutation in exon 6 of *CNGA4* in the common ancestor of Delphinoidea (DelTran; and independently in *Lipotes*) or Delphinida (AccTran; reversed in Inioidea). There are also shared inactivating mutations at the family level or above in Delphinidae, Monodontidae + Phocoenidae (AccTran only), Monodontidae, Phocoenidae (DelTran only), Kogiidae, and Platanistidae (Figure 4 and Figure 5; Table 2). Additional shared inactivating mutations occur within Delphinidae. There are also autapomorphic mutations in 28 odontocete species, including nine different ziphiids. Overall, inactivating mutations in the olfactory-specific genes are pervasive across Odontoceti but are insufficient to document inactivation of the olfactory signal transduction cascade in the common ancestor of this clade or in either of the basal branches within Odontoceti. Instead, the pattern of inactivating mutations suggests that the olfactory signal transduction cascade may have been inactivated independently in as many as 13 (AccTran) to 16 (DelTran) different branches. This number could be smaller because of mutational lag, which occurs when inactivating mutations begin to accumulate on branches that post-date the onset of neutral evolution [36]. It is also noteworthy that Jauhal et al. [44] did not find any shared inactivating mutations in the olfactory-specific *RTP1* and *RTP2* genes that they examined in seven species of Delphinida (*Lipotes*, two monodontids, four delphinids). Finally, 11 ziphiids (beaked whales) possess intact copies of all three olfactory-specific genes that were investigated. This finding begs the question of whether these forms still retain the olfactory sense or alternatively evolved anosmia very recently and do not exhibit inactivating mutations because of mutational lag.

The results of selection analyses are also relevant for understanding the timing of olfactory ablation in odontocetes. If olfaction was entirely abandoned in the common ancestor of Odontoceti, then neutral evolution is expected across all branches of the crown clade. By contrast, if different branch categories in Odontoceti (functional, transitional, pseudogenic) have different dN/dS values, and if values for functional branches are below 1.0, then weak purifying selection may have operated on olfactory-specific genes on some crown-odontocete branches. The results of selection analyses suggest that dN/dS values are lowest for branches with intact copies of the three olfactory-specific genes, intermediate for transitional branches that record the first inactivating mutation in one or more of the olfactory-specific genes, and highest for the fully pseudogenic branch category. Values for the intact branches are still elevated relative to non-cetacean and mysticete branches, but are less than 1.0 as might be expected for genes that are still under weak purifying selection. A log-likelihood ratio test was used to compare the estimated dN/dS value of ~0.72 for the odontocete branches that have intact (putatively functional) copies of all three genes to an analysis with a fixed dN/dS value of 1.0 for the putatively functional branches, and the results were statistically significant (*p* = 3.5 × 10^−3^). Thus, the olfactory system may have retained a minor role in the early evolutionary history of some odontocete clades and even in the more recent history of multiple ziphiids.

### 4.2. Positive Selection?

An unexpected result of codeml analyses with the preferred codon frequency model (CF2) is that two of the three olfactory-specific genes (*CNGA2*, *CNGA4*) may have evolved under positive selection on fully pseudogenic branches with an inactivated olfactory signal transduction cascade (*CNGA2*: dN/dS = 1.40; *CNGA4*: dN/dS = 1.46). A log-likelihood ratio test rejects the hypothesis that dN/dS = 1.0 in the fully pseudogenic category (*CNGA2*: *p* = 0.02; *CNGA4*: *p* = 7.5 × 10^−3^). By contrast with *CNGA2* and *CNGA4*, dN/dS values suggest that fully pseudogenic branches for *OMP* have evolved under purifying selection (dN/dS = 0.80), although this value is not significantly different from expectations under the neutral hypothesis where dN/dS = 1.0 (*p* = 0.40).

In the case of an inactivated gene(s) or biochemical pathways whose function is no longer required, what are the possible explanations for dN/dS values that are significantly higher than the expected neutral value of 1.0? One possibility is that a formerly useful gene has become detrimental to fitness. For example, Huelsmann et al. [76] argued that inactivation of melatonin synthesis (*AANAT*, *ASMT*) and receptor (*MTNR1A*, *MTNR1B*) genes may have been a prerequisite for the adaptive evolution of unihemispheric sleep in cetaceans (also see Lopes-Marques et al. [77] and Emerling et al. [78] for a general discussion of the relationship between melatonin pathway gene inactivation and sleep biology in cetaceans). In such cases, a reasonable hypothesis is that amino acid changes that eliminated function may have been favored by natural selection. However, it is less clear why amino acid changes would still be favored by natural selection on fully pseudogenic branches that post-date the occurrence of an inactivating mutation(s) that ablates gene function, unless the inactivating mutations were only associated with minor effects on protein function as may occur with premature stop codons that are only a few codons upstream from the canonical termination codon. In the present study of olfactory signal transduction genes, the majority of the fully pseudogenic branches post-date a significant collection of inactivating mutations. Crown *Platanista* branches post-date inactivating mutations in all three olfactory genes; crown *Kogia* branches post-date a premature stop codon in exon 4 of *CNGA4* and a whole exon deletion of the largest exon (exon 6) of *CNGA2*; and crown Delphinoidea branches post-date a termination codon mutation in *CNGA4* that results in 17 additional amino acids at the carboxyterminal end. Within Delphinoidea, delphinids share an additional inactivating mutation (donor splice site of intron 5) in *CNGA4* and a premature stop codon in exon 4 of *CNGA2*, phocoenids share two premature stop codons (exons 5 and 6) in *CNGA2*, and monodontids share three frameshift indels (exons 3, 6, and 6) in *CNGA2*. Overall, these are significant inactivating mutations in *CNGA2* and *CNGA4* that are expected to ablate protein function and result in neutral evolution rather than positive selection in the fully pseudogenic category. Given the large numbers of inactivating mutations on both transitional branches (e.g., stem *Platanista*, stem *Kogia*, stem Delphinida or Delphinoidea) and other deep branches within Delphinoidea, something other than positive selection may be required to explain the elevated dN/dS values that were estimated for the fully pseudogenic category. One possibility is that CpG sites, which have elevated substitution rates from C to T when cytosine is methylated, are disproportionately located at nonsynonymous sites.

To test this hypothesis, all codons in the *CNGA2* and *CNGA4* alignments that include all or part of a CpG dinucleotide in the ancestral odontocete were eliminated, and codeml analyses were re-run with the preferred (CF2) codon frequency model. In both cases the resulting dN/dS values for the fully pseudogenic category are not significantly different from the results of analyses with dN/dS fixed at 1.0 in likelihood ratio tests (*p* = 0.37 for *CNGA2*, *p* = 0.06 for *CNGA4*). An additional explanation for the elevated dN/dS values in the fully pseudogenic category is that synonymous codon substitutions are not always selectively neutral [79,80]. For example, Hellmann et al. [81] found that as many as 39% of fourfold degenerate synonymous sites in human protein-coding genes are under purifying selection and do not evolve neutrally. If some synonymous substitutions are under purifying selection, the global synonymous substitution rate that is estimated by codeml will be less than the neutral substitution rate and dN/dS estimates will be inflated. Selection on synonymous substitutions may be the result of synonymous codon usage that varies among different genes and is correlated with gene expression levels [80]. Codon usage bias can also be impacted by other variables such as GC content, recombination rates, RNA stability, and gene length [82]. However, codon bias should not be a factor following gene inactivation, in particular when the mutated sequence no longer encodes a functional protein.

### 4.3. Incomplete Lineage Sorting

Several inactivating mutations have most parsimonious reconstructions that are homoplastic (Figure 4 and Figure 5; Table 2). Homoplastic mutations may be the result of independent gains and/or losses in different lineages or incomplete lineage sorting. In the case of premature stop codons, homoplasy may result from single base substitutions (e.g., TGG to TGA). Other homoplastic inactivating mutations, such as the intron–exon boundary deletion in *CNGA2* that includes the last 7 bp of intron 1 and the first 7 bp of exon 2, seem less likely to arise independently in different lineages given that indels, especially longer indels, occur much less frequently than single-base substitutions [83]. Parsimony reconstructions suggest that the intron–exon boundary deletion in *CNGA2* originated independently in *Orcaella* and *Feresa* + *Peponocephala* + *Globicephala*, but an alternate explanation is that this mutation originated as an allelic variant in the common ancestor of Globicephalinae (pilot whales and kin) and was subsequently lost in three globicephaline sublineages (*Steno bredanensis*, *Grampus griseus*, *Pseudorca crassidens*) that retain an intact intron–exon boundary, in which case it would be an example of hemiplasy rather than homoplasy [84] (p. 504). It is also possible that some of these globicephalines are polymorphic for this mutation given that only one individual was sampled for each species.

### 4.4. Integrating Molecular Results with the Fossil Record

Farnkopf et al. [26] concluded that degeneration of the olfactory system commenced in the Eocene in archaeocete (stem) cetaceans based on their estimates for the reduced number of intact OR genes in a remingtonocetid (307–333 OR genes) from 42 million years ago versus a pakicetid (731–766 OR genes) from 50 million years ago. In rough agreement with this finding, the dN/dS estimate for the stem cetacean branch (~0.28, three olfactory genes) suggests that purifying selection was slightly relaxed relative to selection in terrestrial cetartiodactyls (~0.11). Within crown Cetacea, the dN/dS value for mysticete branches (~0.31) is similar to that for the stem Cetacea branch. This result suggests that purifying selection was maintained at approximately the same level in Mysticeti as on the stem Cetacea branch. This finding is also consistent with the retention of a functional, albeit reduced, olfactory system in baleen whales, as suggested by recent anatomical and gene expression studies [16,17,18,26]. Patterns of gene inactivation and the results of selection analyses on single-copy, olfactory signal transduction genes both support the hypothesis that the most recent common ancestor of crown Odontoceti retained a reduced olfactory sense that was fully eliminated in several independent lineages of the crown clade. Eleven ziphiid species retain intact copies of all three genes, and dN/dS values on fully pseudogenic branches are almost twice as high as those on intact branches within Odontoceti. Overall, these results are consistent with reports of osteological proxies for olfaction that are found in both stem and crown fossil odontocetes. Among stem odontocetes, *Xenorophus* sp. (late Oligocene) and *Prosqualodon davidis* (early Miocene) both retain a small olfactory fossa, which is a depression in the skull that houses the olfactory bulbs [37]. Another stem odontocete, *Agorophius pygmaeus*, retained a strong olfactory sense based on the presence of well-preserved ethmoid labyrinths and cribriform plate [85]. The stem odontocete *Squalodon* possessed well-developed olfactory anatomy, including ethmoturbinates [13], and Farnkopf et al. [26] estimated that *S. murdochi* had 181–203 intact OR genes, which is higher than for any extant odontocete [15,26,39,40]. Small olfactory fossae are also found among putative crown odontocetes including *Xiphiacetus bossi* (early to middle Miocene) and an indeterminate species of Squalodelphinidae (early Miocene) [37,86]. *X. bossi* belongs to the family Eurhinodelphinidae, which is the sister-taxon to Delphinida based on Gaetan et al.’s [38] cladistic analyses. Squalodelphinids, in turn, belong to “Platanistoidea” and are most closely related to *Platanista* among living odontocetes [38]. According to Godfrey [13], representatives of Squalodontidae (stem Odontoceti), Squalodelphinidae (crown Odontoceti), Platanistidae (crown Odontoceti), and Eurhinodelphidae (crown Odontoceti) from the Miocene Chesapeake Group still possess osteological proxies that are indicative of olfactory ability. These include ethmoturbinates with olfactory recesses, a cribriform plate, and olfactory bulb chambers. Geisler et al. [87] reported that *Meherrinia isoni*, a putative late Miocene inioid, retained ovoid depressions that may have held olfactory bulbs or the remnants of these structures. There are also foramina of various sizes that pass through the cribriform plate that may have transmitted branches of the olfactory nerves [87]. Taken together, the reports of anatomical proxies for olfaction in putative stem taxa for Inioidea, Delphinida, and Platanistidae are consistent with patterns of independent inactivation of single-copy, olfactory-specific genes in these clades.

Additional studies of the cranial anatomy of both fossil and extant odontocetes should prove invaluable in elucidating the details of olfactory ablation in Odontoceti. Indeed, there is almost no information on olfactory structures (or lack thereof) in fossil and extant ziphiids. Flower [25] (p. 220) suggested that a very small hole in *Berardius* that “passes through the posterior lateral expansion of the mesethmoid which corresponds to the cribriform plate of other mammals, to the nasal passage, may be an olfactory foramen.” In the case of delphinids, Bird et al. [9] reported that the ethmoid bone of *Tursiops truncatus* has paired foramina that connect the nasal passage to the brain, although these authors did not validate if there are olfactory sensory neurons or a cribriform plate. Farnkopf et al. [26] provided evidence from CT scans that there is a cribriform plate with foramina in four extant odontocetes (*Delphinapterus leucas*, *Kogia breviceps*, *Phocoena phocoena*, *T. truncatus*). Even if there are olfactory sensory neurons in any of these taxa, it seems unlikely that they would remain functional given that there are inactivating mutations in one or more of the olfactory-specific genes that were investigated. In the case of *T. truncatus*, there are two inactivating mutations in *CNGA2* and three inactivating mutations in *CNGA4*. Among the other taxa, *D. leucas* has one inactivating mutation in *CNGA4* and five inactivating mutations in *CNGA2*, *K. breviceps* exhibits two inactivating mutations in *CNGA4* and four inactivating mutations in *CNGA2*, and *P. phocoena* possesses two inactivating mutations in *CNGA4* and three inactivating mutations in *CNGA2*. In a prior comparative analysis of *OMP*, the sampled *P. phocoena* specimen was heterozygous for a modified start codon mutation [36]. Farnkopf et al. [26] suggested *D. leucas* may retain an olfactory sense and noted correctly that no inactivating mutations are present in the *OMP* gene of this taxon [36]. However, the presence of inactivating mutations in two of the critical olfactory signal transduction genes in *D. leucas* weakens any arguments for the retention of an olfactory sense in this species. García de los Ríos y Loshuertos et al. [88] also presented results that are relevant to the question of whether some extant odontocete species may retain an olfactory sense. Specifically, these authors examined the nasal mucosa of three delphinids (*Delphinus delphis*, *Stenella coeruleoalba*, *Globicephala melas*) and reported that olfactory cells were not found during histological analysis. By contrast, Behrmann [89] reported the presence of olfactory cells that cover ethmoturbinates in *P. phocoena*. Behrmann [85] further suggested that toothed whales could have an olfactory sense, as previously hypothesized by Gruhl [90]. A restricted set of odors may be transmitted by branches of the trigeminal nerve rather than the absent olfactory nerve, as occurs in humans whose olfactory nerves have been destroyed, i.e., the detection of odors of nitrate, ammonia, and sal ammoniac [89]. As noted above, inactivating mutations in the *CNGA2* and *CNGA4* gene in *P. phocoena* [36] cast doubt on the functionality of an olfactory system in this taxon that relies on standard molecular components of the olfactory signal transduction cascade. Also, Behrmann’s [89] conclusion that there are ethmoturbinates in *P. phocoena* is at odds with Ichishima’s [16] conclusion that ethmoturbinates are absent in crown odontocetes based on his examination of a variety of odontocetes. Ichishima [16] did not investigate *P. phocoena* but did examine a different phocoenid, *Neophocaena phocaenoides* (Indo-Pacific finless porpoise).

### 4.5. Evidence for Odontocete Olfaction Based on Behavioral Experiments

The possibility of olfaction in some extant odontocetes is further compounded by behavioral experiments on *Tursiops truncatus*, which suggested that bottlenose dolphins were able to discriminate between the airborne odors that emanated from visually equivalent containers with rancid fish versus no fish, both of which were placed along the edge of a pool [91]. One of the odors of rancid fish is generated by trimethylamine. This odorant is detected by TAAR5 [92], which is encoded by one of the genes in the trace amine-associated receptor family. Like OR genes, TAAR genes are primarily expressed in the olfactory epithelium of the main olfactory system that also expresses OR genes in mouse models, although TAAR expression seems to be limited to a distinct type of olfactory sensory neuron that is biased to express TAAR rather than OR genes [93]. TAARs transduce their ligands using the canonical olfactory signal transduction cascade that is employed by ORs [93]. Thus, inactivating mutations in *CNGA2* and *CNGA4* should also impact TAAR signal transduction. Moreover, *TAAR5* is lost in two cetaceans (bottlenose dolphin, Antarctic minke whale) that have been investigated [20]. An alternate explanation for Kremers et al.’s [91] findings is that some of the airborne odors of rancid fish are water soluble and were more concentrated in water that was closer to the container with the rancid fish. Water-soluble odors include short-chain fatty acids (e.g., butyric acid), which can be detected by gustatory senses [94]. Among fatty acid receptors that are associated with taste, free fatty acid receptor 2 (*FFAR2*) and free fatty acid receptor 4 (*FFAR4*) have been accepted as short-chain and long-chain fatty acid (SCFA and LCFA) taste receptors, respectively [95,96]. *FFAR2* is intact in all 16 odontocetes that are annotated in NCBI’s RefSeq genomes database (Supplementary Materials). The fidelity of *FFAR2* in odontocetes suggests that short-chain fatty acids from rancid fish may have provided waterborne signals to the gustatory system of the dolphins in Kremers et al.’s [91] behavioral experiments.

### 4.6. Conclusions

Living adult odontocetes that have been investigated are likely anosmic based on the loss or extreme reduction in anatomical structures that are correlated with olfaction including the olfactory nerve, olfactory tract, olfactory bulb, perforated cribriform plate, and ethmoturbinals that are lined by an olfactory epithelium. However, there are also extinct odontocetes that possess anatomical structures that are proxies for a functional sense of smell, and some of these taxa are putatively in crown Odontoceti based on recent cladistic analyses, e.g., [38]. If olfaction was ablated in the common ancestor of Odontoceti, molecular evidence for this loss of olfaction may be expected in the form of inactivating mutations in single-copy, olfactory-specific genes that are shared by all extant odontocetes and/or evidence of consistently neutral evolution in these genes on all lineages of Odontoceti. Conversely, if olfaction remained functional in the most recent common ancestor of Odontoceti, albeit with reduced functionality, then shared inactivating mutations in single-copy, olfactory-specific genes should not be present across all extant members of Odontoceti. Furthermore, these genes should not record a history of neutral evolution across the entire crown clade. These two competing hypotheses were evaluated using three single-copy, olfactory-specific genes (*CNGA2*, *CNGA4*, *OMP*) in 65 odontocete species. Of these, *CNGA2* and *CNGA4* encode subunits of the cyclic nucleotide-gated channels that are critical for the depolarization and relay of action potentials from the olfactory neurons to the olfactory bulb [49]. *OMP*, in turn, encodes a protein that plays a key role in the olfactory signal transduction cascade of mammals where it may regulate the kinetics and termination of olfactory responses [55]. Evaluations of *CNGA2*, *CNGA4*, and *OMP* did not reveal any inactivating mutations that are shared by all odontocetes or either of the two major clades of Odontoceti (Physeteroidea, Synrhina). Inactivating mutations are also absent in 11 extant ziphiid species. Still, mutations may be absent on the stem odontocete branch as well as various branches within Odontoceti because of mutational lags [36]. It is therefore important to also perform selection analyses to determine if different categories of branches in Odontoceti have evolved under different selection pressures. The results of selection analyses suggest that fully pseudogenic odontocete branches have dN/dS values that are almost twice as high as dN/dS values on odontocete branches in which all three single-copy olfactory genes are intact. These selection results, in conjunction with the pattern of inactivating mutations in olfactory-specific genes, are most consistent with the hypothesis that the most recent common ancestor of crown odontocetes had a functional olfactory sense and that the final stages of olfactory ablation occurred convergently in multiple odontocete lineages. Thus, the extremely rare condition of anosmia, which, to the best of our knowledge, only occurs in Odontoceti (and possibly hydrophiin sea snakes [4]) among the tens of thousands of vertebrate species on Earth, has evolved multiple times within this small twig of the overall vertebrate tree.

## Figures and Tables

**Table 1:** Taxon sampling for 65 odontocetes, 14 mysticetes, 26 cetartiodactyl outgroups, and two perissodactyl outgroups.

Odontocetes		
Family	Species	Common Name
Delphinidae	*Cephalorhynchus commersonii* (Lacépède, 1804)	Commerson’s dolphin
Delphinidae	*Cephalorhynchus eutropia* (Gray, 1846)	Black dolphin/Chilean dolphin
Delphinidae	*Cephalorhynchus heavisidii* (Gray, 1828)	Heaviside’s dolphin
Delphinidae	*Cephalorhynchus hectori* (Van Beneden, 1881)	Hector’s dolphin
Delphinidae	*Delphinus delphis bairdii* (Dall, 1873)	Long-beaked common dolphin
Delphinidae	*Delphinus delphis delphis* (Linnaeus, 1758)	Common dolphin
Delphinidae	*Feresa attenuata* (Gray, 1874)	Pygmy killer whale
Delphinidae	*Globicephala macrorhynchus* (Gray, 1846)	Short-finned pilot whale
Delphinidae	*Globicephala melas* (Traill, 1809)	Long-finned pilot whale
Delphinidae	*Grampus griseus* (G. Cuvier, 1812)	Risso’s dolphin
Delphinidae	*Lagenodelphis hosei* (Fraser, 1956)	Fraser’s dolphin
Delphinidae	*Lagenorhynchus albirostris* (Gray, 1846)	White-beaked dolphin
Delphinidae	*Leucopleurus acutus* (Gray, 1828)	Atlantic white-sided dolphin
Delphinidae	*Lissodelphis borealis* (Peale, 1849)	Northern right whale dolphin
Delphinidae	*Lissodelphis peronii* (Lacépède, 1804)	Southern right whale dolphin
Delphinidae	*Orcaella brevirostris* (Owen in Gray, 1866)	Irrawaddy dolphin
Delphinidae	*Orcaella heinsohni* (Beasley, Robertson and Arnold, 2005)	Australian snubfin dolphin
Delphinidae	*Orcinus orca* (Linnaeus, 1758)	Killer whale
Delphinidae	*Peponocephala electra* (Gray, 1846)	Melon-headed whale
Delphinidae	*Pseudorca crassidens* (Owen, 1846)	False killer whale
Delphinidae	*Sagmatias obliquidens* (Gill, 1865)	Pacific white-sided dolphin
Delphinidae	*Sagmatias obscurus* (Gray, 1828)	Dusky dolphin
Delphinidae	*Sousa chinensis* (Osbeck, 1765)	Indo-Pacific humpback dolphin
Delphinidae	*Stenella attenuata* (Gray, 1846)	Pantropical spotted dolphin
Delphinidae	*Stenella clymene* (Gray, 1850)	Clymene dolphin
Delphinidae	*Stenella coeruleoalba* (Meyen, 1833)	Striped dolphin
Delphinidae	*Stenella frontalis* (G. Cuvier, 1829)	Atlantic spotted dolphin
Delphinidae	*Stenella longirostris* (Gray, 1828)	Spinner dolphin
Delphinidae	*Steno bredanensis* (Lesson, 1828)	Rough-toothed dolphin
Delphinidae	*Tursiops aduncus* (Ehrenberg, 1832)	Indo-Pacific bottlenose dolphin
Delphinidae	*Tursiops truncatus* (Montagu, 1821)	Common bottlenose dolphin
Iniidae	*Inia geoffrensis* (Blainville, 1817)	Amazon River dolphin
Kogiidae	*Kogia breviceps* (Blainville, 1838)	Pygmy sperm whale
Kogiidae	*Kogia sima* (Owen, 1866)	Dwarf sperm whale
Lipotidae	*Lipotes vexillifer* (Miller, 1918)	Chinese river dolphin/baiji
Monodontidae	*Delphinapterus leucas* (Pallas, 1776)	Beluga
Monodontidae	*Monodon monoceros* (Linnaeus, 1758)	Narwhal
Phocoenidae	*Neophocaena asiaeorientalis* (Pilleri and Gihr, 1972)	Yangtze finless porpoise
Phocoenidae	*Neophocaena phocaenoides* (G. Cuvier, 1829)	Indo-Pacific finless porpoise
Phocoenidae	*Phocoena phocoena* (Linnaeus, 1758)	Harbor porpoise
Phocoenidae	*Phocoena sinus* (Norris and McFarland, 1958)	Vaquita
Physeteridae	*Physeter macrocephalus* (Linnaeus, 1758)	Sperm whale
Platanistidae	*Platanista gangetica* (Lebeck, 1801)	Ganges River dolphin
Platanistidae	*Platanista minor* (Owen, 1853)	Indus River dolphin
Pontoporiidae	*Pontoporia blainvillei* (Gervais and d’Orbigny, 1844)	Franciscana/La Plata dolphin
Ziphiidae	*Berardius arnuxii* (Duvernoy, 1851)	Arnoux’s beaked whale
Ziphiidae	*Berardius minimus* (Yamada, Kitamura and Matsuishi, 2019)	Sato’s beaked whale
Ziphiidae	*Hyperoodon ampullatus* (Forster in Kalm, 1770)	Northern bottlenose whale
Ziphiidae	*Hyperoodon planifrons* (Flower, 1882)	Southern bottlenose whale
Ziphiidae	*Indopacetus pacificus* (Longman, 1926)	Tropical bottlenose whale
Ziphiidae	*Mesoplodon bidens* (Sowerby, 1804)	Sowerby’s beaked whale
Ziphiidae	*Mesoplodon bowdoini* (Andrews, 1908)	Andrew’s beaked whale
Ziphiidae	*Mesoplodon carlhubbsi* (Moore, 1963)	Hubb’s beaked whale
Ziphiidae	*Mesoplodon densirostris* (Desmarest, 1817)	Blainville’s beaked whale
Ziphiidae	*Mesoplodon europaeus* (Gervais, 1855)	Gervais’ beaked whale
Ziphiidae	*Mesoplodon ginkgodens* (Nishiwaki and Kamiya, 1958)	Ginkgo-toothed beaked whale
Ziphiidae	*Mesoplodon grayi* von (Haast, 1876)	Gray’s beaked whale
Ziphiidae	*Mesoplodon hectori* (Gray, 1871)	Hector’s beaked whale
Ziphiidae	*Mesoplodon layardii* (Gray, 1865)	Strap-toothed whale
Ziphiidae	*Mesoplodon mirus* (True, 1913)	True’s beaked whale
Ziphiidae	*Mesoplodon perrini* (Dalebout, Mead, Baker, Baker and van Helden, 2002)	Perrin’s beaked whale
Ziphiidae	*Mesoplodon peruvianus* (Reyes, Mead and Van Waerebeek, 1991)	Pygmy beaked whale
Ziphiidae	*Mesoplodon stejnegeri* (True, 1885)	Stejneger’s beaked whale
Ziphiidae	*Tasmacetus shepherdi* (Oliver, 1937)	Shepherd’s beaked whale
Ziphiidae	*Ziphius cavirostris* (G. Cuvier, 1823)	Cuvier’s beaked whale
Mysticeti		
Balaenidae	*Balaena mysticetus* (Linnaeus, 1758)	Bowhead whale
Balaenidae	*Eubalaena australis* (Desmoulins, 1822)	Southern right whale
Balaenidae	*Eubalaena glacialis* (Müller, 1776)	North Atlantic right whale
Balaenidae	*Eubalaena japonica* (Lacépède, 1818)	North Pacific right whale
Balaenopteridae	*Balaenoptera acutorostrata* (Lacépède, 1804)	Common minke whale
Balaenopteridae	*Balaenoptera bonaerensis* (Burmeister, 1867)	Antarctic minke whale
Balaenopteridae	*Balaenoptera borealis* (Lesson, 1828)	Sei whale
Balaenopteridae	*Balaenoptera musculus* (Linnaeus, 1758)	Blue whale
Balaenopteridae	*Balaenoptera omurai* (Wada, Oishi and Yamada, 2003)	Omura’s whale
Balaenopteridae	*Balaenoptera physalus* (Linnaeus, 1758)	Fin whale
Balaenopteridae	*Balaenoptera ricei* (Rosel, Wilcox, Yamada and Mullin, 2021)	Rice’s whale
Balaenopteridae	*Megaptera novaeangliae* (Borowski, 1781)	Humpback whale
Eschrichtiidae	*Eschrichtius robustus* (Lilljeborg, 1861)	Gray whale
Neobalaenidae	*Caperea marginata* (Gray, 1846)	Pygmy right whale
Cetartiodactyl Outgroups		
Antilocapridae	*Antilocapra americana* (Ord, 1815)	Pronghorn
Bovidae	*Aepyceros melampus* (Lichtenstein, 1812)	Impala
Bovidae	*Bos taurus* (Linnaeus, 1758)	Wild yak
Bovidae	*Bubalus bubalis* (Linnaeus, 1758)	Water buffalo
Bovidae	*Capra hircus* (Linnaeus, 1758)	Domestic goat
Bovidae	*Cephalophus harveyi* (Thomas, 1893)	Harvey’s red duiker
Bovidae	*Connochaetes taurinus* (Burchell, 1824)	Blue wildebeest
Bovidae	*Kobus leche* (Gray, 1850)	Lechwe
Bovidae	*Litocranius walleri* (Brooke, 1879)	Gerenuk
Bovidae	*Nanger granti* (Brooke, 1872)	Grant’s gazelle
Bovidae	*Neotragus pygmaeus* (Linnaeus, 1758)	Royal antelope
Bovidae	*Oreotragus oreotragus* (Zimmermann, 1783)	Klipspringer
Bovidae	*Oryx dammah* (Cretzschmar, 1827)	Scimitar oryx
Bovidae	*Ovis aries* (Linnaeus, 1758)	Domestic sheep
Bovidae	*Tragelaphus eurycerus* (Ogilby, 1837)	Bongo
Camelidae	*Camelus ferus* (Przewalski, 1883)	Wild Bactrian camel
Camelidae	*Vicugna pacos* (Linnaeus, 1758)	Alpaca
Cervidae	*Axis porcinus* (Zimmermann, 1780)	Indian hog deer
Cervidae	*Odocoileus virginianus* (Zimmermann, 1780)	White-tailed deer
Giraffidae	*Giraffa camelopardalis* (Linnaeus, 1758)	Giraffe
Giraffidae	*Okapia johnstoni* (P. L. Sclater, 1851)	Okapi
Hippopotamidae	*Hippopotamus amphibius* (Linnaeus, 1758)	River hippopotamus
Moschidae	*Moschus moschiferus* (Linnaeus, 1758)	Siberian musk deer
Suidae	*Sus scrofa* (Linnaeus, 1758)	Wild boar
Tayassuidae	*Catagonus wagneri* (Rusconi, 1930)	Chacoan peccary
Tragulidae	*Tragulus javanicus* (Osbeck, 1765)	Java mouse-deer
Perissodactyl outgroups		
Rhinocerotidae	*Ceratotherium simum* (Burchell, 1817)	White rhinoceros
Equidae	*Equus caballus* (Linnaeus, 1758)	Horse

**Table 2:** Most parsimonious mapping of inactivating mutations in odontocete olfactory genes based on DelTran character optimization, which depicts the maximum number of mutations. In cases of equivocal resolutions between AccTran and DelTran character optimization, AccTran reconstructions are described in the footnotes below.

Odontoceti Taxon	Olfactory Gene		
	*CNGA2*	*CNGA4*	*OMP*
*Physeter*	265I (E3), I4-E5BD (52 bp of I4 and 6 bp of E5), 648–649I (E6), 674–683D (E6), 698–874D (E6), 1612–1645D (E6)		1–3SCM (E1)
*Kogia*	222I (E3), 604–2019WED (E6)	339–341S (E4)	
*Kogia breviceps*	1–3SCM (CTG, E1), 444–465D (E4)	In5Do (AT)	
*Kogia sima*	1–3SCM (AGG, E1), 445–467D (E4)		445D (E1)
*Platanista*	440–442S (E4) ^b^, 1761–1763S (E6)	1158–1160S	89–90D (E1)
*Indopacetus pacificus*		918–920S (E4), 1006D (E5)	
*Mesoplodon bidens*	591–593S (E5), 830I (E6)	In1Do (TT), 843–844D (E4)	
*Mesoplodon bowdoini*		1151I (E5)	
*Mesoplodon carlhubbsi*	440–442S (E4) ^b^		
*Mesoplodon densirostris*	284–300D (E3)	1183–1185S (E5)	
*Mesoplodon europaeus*	1062–1064S (E6)	92–93I (E2)	
*Mesoplodon hectori*			306–312D (E1)
*Mesoplodon perrini*	404–405D (E4)		318–337I (E1)
*Mesoplodon stejnegeri*	176D (E2)		125I (E1)
*Lipotes vexillifer*		529–531S (E4), 768D (E4), 1314–1315I (E6), 1747–1749TCM (GGA, E6) ^g^	461D (E1)
*Inia geoffrensis*	451D (E4), 888–890S (E6), 897–899S (E6), 1809–1811S (E6)	In3Ac (GG), In5Do (GG)	1–3SCM (E1), 6D (E1)
*Pontoporia blainvilllei*	E1-I1BD (37 bp of E1 and 28 bp of I1), In4Ac (AA), 984–986S (E6), 1638–1640S (E6) ^f^	3–9D (E1), 1417I (E6), 1723–1725S (E6)	40I (E1)
Delphinida		1747–1749TCM (GGA, E6) ^g^	
Monodontidae	370–374I (E3), 1176–1641D (E6), 1961–1964I (E6)		
*Delphinapterus leucas*	674–676S (E6) ^e^, 1684D (E6)		
*Monodon monoceros*	501–503S (E5) ^d^, 222I (E3)	879I (E4)	
Phocoenidae	501–503S (E5) ^d^, 674–676S (E6) ^e^		
*Phocoena phocoena*	682–752D (E6)	1558–1560S (E6)	
*Phocoena sinus*		1132–1133D (E5)	
Delphinidae	440–442S (E4)	In5Do (GA)	
*Leucopleurus acutus*	348D (E3), 501–503S (E5) ^d^, 1046–1049D (E6), 1638–1640S (E6) ^f^	413–416I (E4) ^i^, 1403D (E1)	
All delphinids except *Leucopleurus acutus*	1458–1467D (E6)		
All delphinids except *Leucopleurus acutus* and *Orcinus orca*		306D (E4) ^h^	
*Orcinus orca*		413–416I (E4) ^i^	
*Lagenorhynchus albirostris*	1044D (E6)		
*Lissodelphis* + *Sagmatias* + *Cephalorhynchus*	591–593S (E5)		
*Lissodelphis*	1557–1559S (E6)		
*Lissodelphis peronii*	487I (E4)		
*Sagmatias obliquidens*			107I (E1)
*Cephalorhynchus hectori* + *C. commersonii* + *C. eutropia*		537–543I (E4)	
*Orcaella* + *Steno* + *Grampus* + *Pseudorca* + *Feresa* + *Peponocephala* + *Globicephala*		413–416I (E4) ^i^	
*Orcaella*	I1-E2BD (7bp of I1 and 7bp of E2) ^a^		
*Steno bredanensis*		1732D (E6)	
*Feresa* + *Peponocephala* + *Globicephala*	I1-E2BD (7bp of I1 and 7bp of E2) ^a^		
*Feresa* + *Peponocephala*	1620–1622S (E6)		
*Feresa attenuata*		218D (E3)	
*Globicephala*	E3-I3BD (14 bp of E3 and 30 bp of I3)	30–31D (E1)	
*Sousa chinensis*	501–503S (E5) ^d^		
*Stenella attenuata* + *S. frontalis*	214–216S (E3) ^c^, 501–503S (E5) ^d^		
*Stenella frontalis*	1638–1640S (E6)		
*Stenella coeruleoalba*	214–216S (E3) ^c^		
*Stenella clymene* + *S. longirostris*	501–503S (E5) ^d^		
*Stenella clymene*	214–216S (E3) ^c^		

^a^ Homoplastic boundary deletion in *Orcaella* and *Feresa* + *Peponocephala* + *Globicephala* with both AccTran and Deltran. ^b^ Homoplastic premature stop codon (TGA) in *Platanista* and *Mesoplodon carlhubbsi* with both AccTran and Deltran. Delphinids have a TAG stop codon at the same position. ^c^ Homoplastic premature stop codon (TGA) in *Stenella clymene*, *S. coeruleoalba*, and *S. attenuata* + *S. frontalis* with both AccTran and DelTran. ^d^ Homoplastic premature stop codon (TGA) in Phocoenidae, *Monodon monoceros*, *Leucopleurus acutus*, *Sousa chinensis*, *Stenella attenuata* + *S. frontalis*, and *S. clymene* + *S. longirostris*. With AccTran character optimization, the premature stop codon is gained in Phocoenidae + Monodontidae and then lost in *Delphinapterus leucas*. ^e^ Homoplastic premature stop codon in Phocoenidae and *Delphinapterus leucas*. With AccTran character optimization, the premature stop codon is gained in Phocoenidae + Monodontidae and then lost in *Monodon monoceros*. ^f^ Homoplastic premature stop codon (TGA) in *Leucopleurus acutus* and *Pontoporia blainvillei* with both AccTran and Deltran. ^g^ Homoplastic termination codon mutation in Delphinida and *Lipotes vexillifer* with DelTran character optimization. AccTran character optimization also requires two steps (gain in Delphinida + *Lipotes* + Inioidea with reversal in Inioidea). ^h^ Deletion is reversed in *Orcaella* + *Steno* + *Grampus* + *Pseudorca* + *Feresa* + *Peponocephala* + *Globicephala* with both AccTran and DelTran. ^i^ Homoplastic frameshift insertion in *Leucopleurus acutus*, *Orcinus orca*, and *Orcaella* + *Steno* + *Grampus* + *Pseudorca* + *Feresa* + *Peponocephala* + *Globicephala* with both AccTran and DelTran. Numbers correspond to positions in the protein-coding sequence alignments (CDS). Abbreviations: Ac = acceptor splice site; D = frameshift deletion; Do = donor splice site; E = exon; I = frameshift insertion; In = intron; S = premature stop codon; SCM = start codon mutation; TCM = termination codon mutation; WED = whole exon deletion.

**Table 3:** Results of dN/dS analyses with six branch categories and all codon sites.

Branch Category	*CNGA2*	*CNGA4*	*OMP*	3 Genes
Non-cetacean	0.0952	0.1019	0.1318	0.1113
Stem Cetacea	0.2402	0.3189	0.1843	0.2781
Mysticeti	0.4531	0.2418	0.1348	0.3067
Intact Odontoceti	0.8187	0.7118	0.3477	0.7165
Transitional Odontoceti	1.1429	0.8746	0.4807	0.9538
Fully Pseudogenic	1.3994	1.4558	0.7960	1.3871

## Data Availability

Data are contained within the article and the Supplementary Materials.

## Supplementary Materials

The following supporting information can be downloaded at: https://www.mdpi.com/article/10.3390/genes17060651/s1, (1) alignments for protein-coding sequences (CDS) for *CNGA2*, *CNGA4*, and *OMP*; (2) maximum likelihood (RAxML) gene trees with bootstrap support values in Newick format; (3) files for codeml analyses with PAML; (4) *FFAR2* coding sequence alignment for 16 odontocetes; (5) accession numbers for olfactory gene sequences used in this study and sample ID information for newly generated sequences (Table S1); and (6) Akaike Information Criterion tests (Table S2).

## References

[1] Poncelet G., Shimeld S.M. (2020). The evolutionary origins of the vertebrate olfactory system. Open Biol..

[2] Niimura Y. (2012). Olfactory receptor multigene family in vertebrates: From the viewpoint of evolutionary genomics. Curr. Genom..

[3] Korsching S.I. (2025). Evolution of vertebrate olfactory receptor repertoires and their function. Curr. Opin. Behav. Sci..

[4] Kishida T. (2021). Olfaction of aquatic amniotes. Cell Tissue Res..

[5] Meyer A., Schloissnig S., Franchini P., Du K., Woltering J.M., Irisarri I., Wong W.Y., Nowoshilow S., Kneitz S., Kawaguchi A. (2021). Giant lungfish genome elucidates the conquest of land by vertebrates. Nature.

[6] Niimura Y. (2009). On the origin and evolution of vertebrate olfactory receptor genes: Comparative genome analysis among 23 chordate species. Genome Biol. Evol..

[7] Rowe T.B., Macrini T.E., Luo Z.-X. (2011). Fossil evidence on origin of the mammalian brain. Science.

[8] Niimura Y., Matsui A., Touhara K. (2014). Extreme expansion of the olfactory receptor gene repertoire in African elephants and evolutionary dynamics of orthologous gene groups in 13 placental mammals. Genome Res..

[9] Bird D.J., Murphy W.J., Fox-Rosales L., Hamid I., Eagle R.A., Van Valkenburgh B. (2018). Olfaction written in bone: Cribriform plate size parallels olfactory receptor gene repertoires in Mammalia. Proc. Roy. Soc. B.

[10] Zhang T., Jing H., Wang J., Zhao L., Liu Y., Rossiter S.J., Lu H., Li G. (2024). Evolution of olfactory receptor superfamily in bats based on high throughput molecular modeling. Mol. Ecol. Resour..

[11] Boesveldt S., Postma E.M., Boak D., Welge-Luessen A., Schöpf V., Mainland J.D., Martens J., Ngai J., Duffy V. (2017). Anosmia—A clinical review. Chem. Senses.

[12] Oleszkiewicz A., Croy I., Hummel T. (2025). The impact of olfactory loss on quality of life: A 2025 review. Chem. Senses.

[13] Godfrey S.J. (2013). On the olfactory apparatus in the Miocene odontocete *Squalodon* sp. (Squalodontidae). Comptes Rendus Palevol.

[14] Berta A., Ekdale E.G., Cranford T.W. (2014). Review of the cetacean nose: Form, function, and evolution. Anat. Rec..

[15] Buono M., Fernández M.S., Fordyce R.E., Reidenberg J.S. (2015). Anatomy of nasal complex in the southern right whale, *Eubalaena australis* (Cetacea, Mysticeti). J. Anat..

[16] Ichishima H. (2016). The ethmoid and presphenoid of cetaceans. J. Morphol..

[17] Farnkopf I.C., George J.C., Kishida T., Hillmann D.J., Suydam R.S., Thewissen J.G.M. (2022). Olfactory epithelium and ontogeny of the nasal chambers in the bowhead whale (*Balaena mysticetus*). Anat. Rec..

[18] Hirose A., Nakamura G., Nikaido M., Fujise Y., Kato H., Kishida T. (2024). Localized expression of olfactory receptor genes in the olfactory organ of common minke whales. Int. J. Mol. Sci..

[19] Thewissen J.G.M., George J., Rosa C., Kishida T. (2011). Olfaction and brain size in the bowhead whale (*Balaena mysticetus*). Mar. Mamm. Sci..

[20] Kishida T., Thewissen J.G.M., Hayakawa T., Imai H., Agata K. (2015). Aquatic adaptation and the evolution of smell and taste in whales. Zool. Lett..

[21] Cave A.J.E. (1988). Note on olfactory activity in mysticetes. J. Zool. Lond..

[22] Thewissen J.G.M., Würsig B., Thewissen J.G.M., Kovacs K.M. (2018). Sensory biology. Encyclopedia of Marine Mammals.

[23] Mead J.G., Fordyce R.E. (2009). The therian skull: A lexicon with emphasis on the odontocetes. Smithson. Contrib. Zool..

[24] Flower W.H. (1868). On the osteology of the cachalot or sperm-whale (*Physeter macrocephalus*). Trans. Zool. Soc. Lond..

[25] Flower W.H. (1872). On the recent ziphioid whales, with a description of the skeleton of *Berardius arnouxi*. Trans. Zool. Soc. Lond..

[26] Farnkopf I., Kishida T., Sato Y., Reidenberg J.S., Niimura Y., Thewissen J.G.M. (2025). Cribriform plate size documents loss of olfactory receptor genes in the early evolution of whales, dolphins, and porpoises. Zool. J. Linn. Soc..

[27] Oelschläger H.A., Buhl E.H. (1985). Development and rudimentation of the peripheral olfactory system in the harbor porpoise *Phocoena phocoena* (Mammalia: Cetacea). J. Morphol..

[28] Buhl E.H., Oelschläger H.A. (1988). Morphogenesis of the brain in the harbour porpoise. J. Comp. Neurol..

[29] Yamagiwa D., Endo H., Nakanishi I., Kusanagi A., Kurohmaru M., Hayashi Y. (1999). Anatomy of the cranial nerve foramina in the Risso’s dolphin (*Grampus griseus*). Ann. Anat..

[30] Oelschläger H.A. (2008). The dolphin brain—A challenge for synthetic neurobiology. Brain Res. Bull..

[31] Kishida T., Thewissen J.G.M. (2012). Evolutionary changes of the importance of olfaction in cetaceans based on the olfactory marker protein gene. Gene.

[32] Geisler J.H., Colbert M.W., Carew J.L. (2014). A new fossil species supports an early origin for toothed whale echolocation. Nature.

[33] Liu Z., Qi F.-Y., Xu D.-M., Zhou X., Shi P. (2018). Genomic and functional evidence reveals molecular insights into the origin of echolocation in whales. Sci. Adv..

[34] Heyning J.E. (1997). Sperm whale phylogeny revisited: Analysis of the morphological evidence. Mar. Mamm. Sci..

[35] Gatesy J., Geisler J.H., Chang J., Buell C., Berta A., Meredith R.W., Springer M.S., McGowen M.R. (2013). A phylogenetic blueprint for a modern whale. Mol. Phylogenet. Evol..

[36] Springer M.S., Gatesy J. (2017). Inactivation of the olfactory marker protein (OMP) gene in river dolphins and other odontocete cetaceans. Mol. Phylogenet. Evol..

[37] Tanaka Y., Ortega M., Fordyce R.E. (2023). A new early Miocene archaic dolphin (Odontoceti, Cetacea) from New Zealand, and brain evolution of the Odontoceti. N. Z. J. Geol. Geophys..

[38] Gaetán C.M., Buono M.R., Gaetano L.C. (2019). *Prosqualodon australis* (Cetacea: Odontoceti) from the early Miocene of Patagonia, Argentina: Redescription and phylogenetic analysis. Ameghiniana.

[39] Han W., Wu Y., Zeng L., Zhao S. (2022). Building the Chordata Olfactory Receptor Database using more than 400,000 receptors annotated by Genome2OR. Sci. China Life Sci..

[40] Han W., Bao S., Liu J., Wu Y., Zeng L., Zhang T., Chen N., Yao K., Fan S., Huang A. (2025). The chordata olfactory receptor database. Protein Cell.

[41] Geisler J.H., McGowen M.R., Yang G., Gatesy J. (2011). A supermatrix analysis of genomic, morphological, and paleontological data from crown Cetacea. BMC Evol. Biol..

[42] Pyenson N.D., Vélez-Juarbe J., Gutstein C.S., Little H., Vigil D., O’Dea A. (2015). *Isthminia panamensis*, a new fossil inioid (Mammalia, Cetacea) from the Chagres Formation of Panama and the evolution of ‘river dolphins’ in the Americas. PeerJ.

[43] Hayden S., Bekaert M., Crider T.A., Mariani S., Murphy W.J., Teeling E.C. (2010). Ecological adaptation determines functional mammalian olfactory subgenomes. Genome Res..

[44] Jauhal A.A., Constantine R., Newcomb R.D. (2024). A comparative genomics approach to understanding the evolution of olfaction in cetaceans. J. Mol. Evol..

[45] Shu D.-G., Luo H.-L., Conway Morris S., Zhang Z.-L., Hu S.-X., Chen L., Han J., Zhu M., Li Y., Chen L.-Z. (1999). Lower Cambrian vertebrates from south China. Nature.

[46] Shu D.-G., Conway Morris S., Han J., Zhang Z.-F., Yasui K., Janvier P., Chen L., Zhang X.-L., Liu J.-N., Li Y. (2003). Head and backbone of the Early Cambrian vertebrate *Haikouichthys*. Nature.

[47] Biel M., Michalakis S. (2007). Function and dysfunction of CNG channels: Insights from channelopathies and mouse models. Mol. Neurobiol..

[48] Michalakis S., Reisert J., Geiger H., Wetzel C., Zong X., Bradley J., Spehr M., Hüttl S., Gerstner A., Pfeifer A. (2006). Loss of CNGB1 protein leads to olfactory dysfunction and subciliary cyclic nucleotide-gated channel trapping. J. Biol. Chem..

[49] Nache V., Wongsamitkul N., Kusch J., Zimmer T., Schwede F., Benndorf K. (2016). CNGB1b subunit in olfactory CNG channels. Sci. Rep..

[50] Brunet L.J., Gold G.H., Ngai J. (1996). General anosmia caused by a targeted disruption of the mouse olfactory cyclic nucleotide-gated cation channel. Neuron.

[51] Sailani M.R., Jingga I., MirMazlomi S.H., Bitarafan F., Bernstein J.A., Snyder M.P., Garshasbi M. (2017). Isolated congenital anosmia and *CNGA2* mutation. Sci. Rep..

[52] Karstensen H.G., Mang Y., Fark T., Hummel T., Tommerup N. (2015). The first mutation in *CNGA2* in two brothers with anosmia. Clin. Genet..

[53] Munger S.D., Lane A.P., Zhong H., Leinders-Zufall T., Yau K.-W., Zufall F., Reed R.R. (2001). Central role of the CNGA4 channel subunit in Ca^2+^-calmodulin-dependent odor adaptation. Science.

[54] Kelliher K.R., Ziesmann J., Munger S.D., Reed R.R., Zufall F. (2003). Importance of the CNGA4 channel gene for odor discrimination and adaptation in behaving mice. Proc. Natl. Acad. Sci. USA.

[55] Reisert J., Yau K.-W., Margolis F.L. (2007). Olfactory marker protein modulates the cAMP kinetics of the odour-induced response in cilia of mouse olfactory receptor neurons. J. Physiol..

[56] Buiakova O.I., Baker H., Scott J.W., Farbman A., Kream R., Grillo M., Franzen L., Richman M., Davis L.M., Abbondanzo S. (1996). Olfactory marker protein (OMP) gene deletion causes altered physiological activity of olfactory sensory neurons. Proc. Natl. Acad. Sci. USA.

[57] Dudchenko O., Batra S.S., Omer A.D., Nyquist S.K., Hoeger M., Durand N.C., Shamim M.S., Machol I., Lander E.S., Aiden A.P. (2017). De novo assembly of the *Aedes aegypti* genome using Hi-C yields chromosome-length scaffolds. Science.

[58] Keane M., Semeiks J., Webb A.E., Li Y.I., Quesada V., Craig T., Madsen L.B., van Dam S., Brawand D., Marques P.I. (2015). Insights into the evolution of longevity from the bowhead whale genome. Cell Rep..

[59] Kearse M., Moir R., Wilson A., Stones-Havas S., Cheung M., Sturrock S., Buxton S., Cooper A., Markowitz S., Duran C. (2012). Geneious basic: An integrated and extendable desktop software platform for the organization and analysis of sequence data. Bioinformatics.

[60] Katoh K., Misawa K., Kuma K., Miyata T. (2002). MAFFT: A novel method for rapid multiple sequence alignment based on fast Fourier transform. Nucleic Acids Res..

[61] Katoh K., Toh H. (2008). Recent developments in the MAFFT multiple sequence alignment program. Brief. Bioinform..

[62] Katoh K., Standley D.M. (2013). MAFFT multiple sequence alignment software version 7: Improvements in performance and usability. Mol. Biol. Evol..

[63] Swofford D.L. (2002). Phylogenetic Analysis Using Parsimony (* and Other Methods).

[64] Stamatakis A. (2014). RAxML Version 8: A tool for phylogenetic analysis and postanalysis of large phylogenies. Bioinformatics.

[65] McGowen M.R., Tsagkogeorga G., Álvarez-Carretero S., Dos Reis M., Struebig M., Deaville R., Jepson P.D., Jarman S., Polanowski A., Morin P.A. (2020). Phylogenomic resolution of the cetacean tree of life using target sequence capture. Syst. Biol..

[66] Meredith R.W., Janecka J.E., Gatesy J., Ryder O.A., Fisher C.A., Teeling E.C., Goodbla A., Eizirik E., Simao T.L.L., Stadler T. (2011). Impacts of the Cretaceous Terrestrial Revolution and KPg extinction on mammal diversification. Science.

[67] Hassanin A., Delsuc F., Ropiquet A., Hammer C., Jansen Van Vuuren B., Matthee C., Ruiz-Garcia M., Catzeflis F., Areskoug V., Nguyen T.T. (2012). Pattern and timing of diversification of Cetartiodactyla (Mammalia, Laurasiatheria), as revealed by a comprehensive analysis of mitochondrial genomes. Comptes Rendus Biol..

[68] Rosel P.E., Wilcox L.A., Yamada T.K., Mullin K.D. (2021). A new species of baleen whale (*Balaenoptera*) from the Gulf of Mexico, with a review of its geographic distribution. Mar. Mammal Sci..

[69] McGowen M.R., Spaulding M., Gatesy J. (2009). Divergence date estimation and a comprehensive molecular tree of extant cetaceans. Mol. Phylogenet. Evol..

[70] Zurano J.P., Magalhães F.M., Asato A.E., Silva G., Bidau C.J., Mesquita D.O., Costa G.C. (2019). Cetartiodactyla: Updating a time-calibrated molecular phylogeny. Mol. Phylogenet. Evol..

[71] Yamada T.K., Kitamura S., Abe S., Tajima Y., Matsuda A., Mead J.G., Matsuichi T.F. (2019). Description of a new species of beaked whale (*Berardius*) found in the North Pacific. Sci. Rep..

[72] Chehida Y.B., Thumloup J., Schumacher C., Harkins T., Aguilar A., Borrell A., Ferreira M., Rojas-Bracho L., Robertson K.M., Taylor B.L. (2020). Mitochondrial genomics reveals the evolutionary history of the porpoises (Phocoenidae) across the speciation continuum. Sci. Rep..

[73] Yang Z. (2007). PAML 4: Phylogenetic analysis by maximum likelihood. Mol. Biol. Evol..

[74] Elanga N., Kim S.-H., Vigoda E., Yi S.V., NISC Comparative Sequencing Program (2008). Mutations of different molecular origins exhibit contrasting patterns of regional substitution rate variation. PLoS Comput. Biol..

[75] Springer M.S., Gatesy J. (2016). The gene tree delusion. Mol. Phylogenet. Evol..

[76] Huelsmann M., Hecker N., Springer M.S., Gatesy J., Sharma V., Hiller M. (2019). Genes lost during the transition from land to water in cetaceans highlight genomic changes associated with aquatic adaptations. Sci. Adv..

[77] Lopes-Marques M., Ruivo R., Alves L.Q., Sousa N., Machado A.M., Castro L.F.C. (2019). The singularity of Cetacea behavior parallels the complete inactivation of melatonin gene modules. Genes.

[78] Emerling C.A., Springer M.S., Gatesy J., Jones Z., Hamilton D., Xia-Zhu D., Collin M., Delsuc F. (2021). Genomic evidence for the parallel regression of melatonin synthesis and signaling pathways in placental mammals. Open Res. Eur..

[79] Rahman S., Pond S.L.K., Webb A., Hey J. (2021). Weak selection on synonymous codons substantially inflates *dN/dS* estimates in bacteria. Proc. Natl. Acad. Sci. USA.

[80] Gu X. (2022). *dN/dS-H*, a new test to distinguish different selection modes in protein evolution and cancer evolution. J. Mol. Evol..

[81] Hellmann I., Zöllner S., Enard W., Ebersberger I., Nickel B., Pääbo S. (2003). Selection on human genes as revealed by comparisons to chimpanzee DNA. Genome Res..

[82] Behura S.K., Severson D.W. (2013). Codon usage bias: Causative factors, quantification methods and genome-wide patterns: With emphasis on insect genomes. Biol. Rev..

[83] Zhang Z., Gerstein M. (2003). Patterns of nucleotide substitution, insertion and deletion in the human genome inferred from pseudogenes. Nucleic Acids Res..

[84] Avise J.C., Robinson T.J. (2008). Hemiplasy: A new term in the lexicon of phylogenetics. Syst. Biol..

[85] Boessenecker R.W., Geisler J.H. (2018). New records of the archaic dolphin *Agorophius* (Mammalia: Cetacea) from the upper Oligocene Chandler Bridge Formation of South Carolina, USA. PeerJ.

[86] Cozzuol M.A., Aguilera O.A. (2008). Cetacean remains from the Neogene of northwestern Venezuela. Paläontologische Z..

[87] Geisler J.H., Godfrey S.J., Lambert O. (2012). A new genus and species of late Miocene inioid (Cetacea, Odontoceti) from the Meherrin River, North Carolina, U.S.A.. J. Vertebr. Paleontol..

[88] García de los Ríos y Loshuertos A., Soler Laguía M., Arencibia Espinosa A., López Fernández A., Covelo Figueiredo P., Martínez Gomariz F., Sánchez Collado C., García Carrillo N., Ramírez Zarzosa G. (2021). Comparative anatomy of the nasal cavity in the common dolphin *Delphinus delphis* L., striped dolphin *Stenella coeruleoalba* M. and pilot whale *Globicephala melas* T.: A developmental study. Animals.

[89] Behrmann G. (1989). The olfactory regions in the nose of the harbour porpoise *Phocoena phocoena* (Linne, 1758). Aquat. Mamm..

[90] Gruhl K. (1911). Beitriige zur anatomie und physiologie der Cetaceennase. Jena. Z. Fiir Naturwissenschaften.

[91] Kremers D., Célérier A., Schaal B., Campagna S., Trabalon M., Böye M., Hausberger M., Lemasson A. (2016). Sensory perception in cetaceans: Part II—Promising experimental approaches to study chemoreception in dolphins. Front. Ecol. Evol..

[92] Li Q., Liberles S.D., Zufall F., Munger S.D. (2016). Odor sensing by trace amine-associated receptors. Chemosensory Transduction: The Detection of Odors, Tastes, and Other Chemostimuli.

[93] Dewan A. (2021). Olfactory signaling via trace amine-associated receptors. Cell Tissue Res..

[94] Shackley M., Ma Y., Tate E.W., Brown A.J.H., Frost G., Hanyaloglu A.C. (2020). Short chain fatty acids enhance expression and activity of the umami taste receptor in enteroendocrine cells via a Ga_i/o_ pathway. Front. Nutr..

[95] Hanselman E.C., Amado N.J., Breslin P.A.S. (2021). Oral signals of short and long chain fatty acids: Parallel taste pathways to identify microbes and triclycerides. Curr. Opin. Physiol..

[96] Fontanini A. (2023). Taste. Curr. Biol..

